# Olefin cross metathesis based de novo synthesis of a partially protected L-amicetose and a fully protected L-cinerulose derivative

**DOI:** 10.3762/bjoc.10.102

**Published:** 2014-05-06

**Authors:** Bernd Schmidt, Sylvia Hauke

**Affiliations:** 1 Institut für Chemie, Organische Synthesechemie, Universität Potsdam, Karl-Liebknecht-Straße 24–25, 14476 Potsdam-Golm, Germany

**Keywords:** carbohydrates, de novo synthesis, lactate, metathesis, ruthenium

## Abstract

Cross metathesis of a lactate derived allylic alcohol and acrolein is the entry point to a de novo synthesis of 4-benzoate protected L-amicetose and a cinerulose derivative protected at C5 and C1.

## Introduction

**Figure 1:**
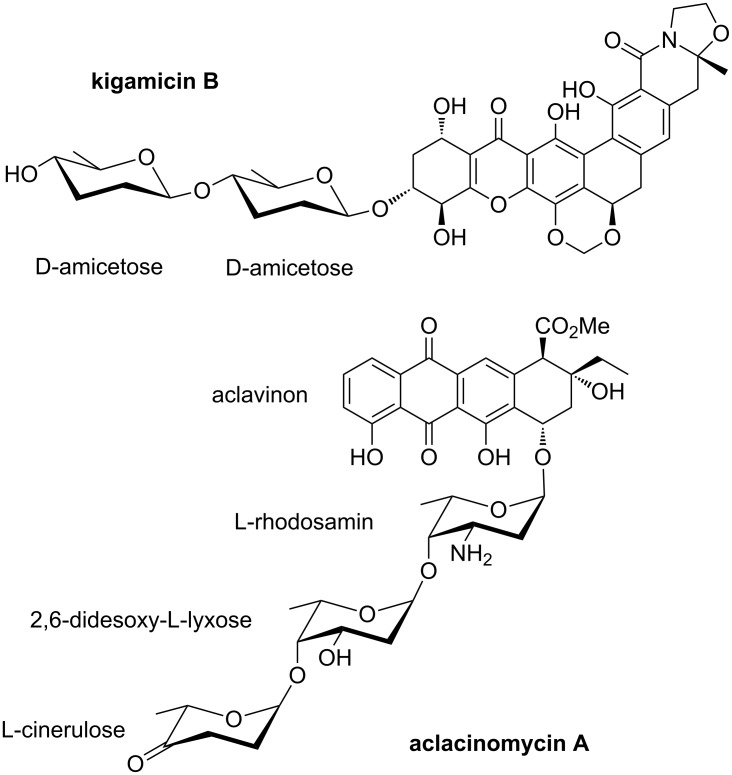
Structures of kigamicin B and aclacinomycin A as representative examples for antineoplastic glycoconjugates.

Many drugs and bioactive natural products are glycoconjugates, which contain an aglycon part linked through glycosidic bonds to one or more oligosaccharide side chains [1]. While it was assumed for quite some time that the carbohydrate side chain merely influences the pharmacokinetics, more recent investigations led to the conclusion that the oligosaccharide moiety contributes essentially to the mechanisms of action, e.g., through molecular recognition of a preferred binding site [2–5], thereby ensuring the selectivity of a chemotherapeutic agent. Particularly common are side chains composed of deoxygenated sugars [6]. For example, the kigamicins are bacterial secondary metabolites isolated from *Amicolatopsis sp*. [7–8] and display both antibiotic and cytotoxic activity [9]. They have a polycyclic xanthone aglycone in common, which is glycosylated at the C14–OH group. In Figure 1 the structure of kigamicin B, which carries a D-amicetose disaccharide unit, is shown as a representative example. Another antitumor antibiotic is aclacinomycin A, which has been used clinically under the name aclarubicin. It is an anthracycline [10] bearing a trisaccharide side chain consisting of L-rhodosamin, 2,6-didesoxy-L-lyxose, and L-cinerulose attached to the aglycon aclavinon [11–12]. It was found to be a potent antineoplastic agent with particular activity against different forms of leukemia [13] (Figure 1).

Desoxy-sugars are sometimes scarcely available from natural sources and therefore the chemical syntheses of the monosaccharides [6,14] and their assembly to oligosaccharides [15–17] has attracted constant attention for many years. For example, D-amicetose has been obtained from other carbohydrates through ethanethiolysis and subsequent Ni-catalyzed desulfurization [18–19], by reduction of the corresponding aldonolactone [20], or by conversion of a protected mannopyranoside into the 2,3-unsaturated enopyranoside and subsequent hydrogenation and reductive 6-deoxygenation [21–22]. Successful de novo approaches [14] to both enantiomers of amicetose include an Achmatowicz rearrangement–hydrogenation sequence, starting from enantiomerically pure 2-(1-hydroxyethyl)furan [23–25], a sequence comprising perdeuteration of an alkynoate derived from L-threonine (giving a 2,2,3,3-tetradeuterated amicetose) [26], enantioselective hydroboration of hetero Diels–Alder adducts [27], stereoconservative diazotation of L-glutamic acid [28–29], diastereoselective addition of methylmagnesium bromide to enantiopure 2-benzyloxyhex-5-enal [30], oxidative degradation of aromatics and subsequent lactonization [31], and a two-carbon homologation of an enantiopure C4-building block with a metallated sulfone [32]. A combinatorial biosynthetic approach to D- and L-amicetose has also recently been established by combining different sugar biosynthesis genes [5].

**Scheme 1:**
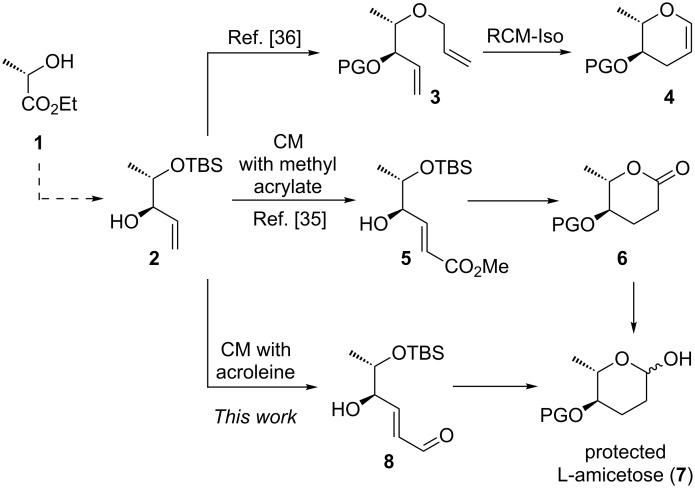
RCM-isomerization approach to L-amicetal **4** and alternative CM approaches to L-amicetose.

Comparatively few transition metal mediated or -catalyzed reactions have hitherto been used for the synthesis of 2,3,6-tridesoxy sugars such as amicetose. An example is the W-promoted cycloisomerization of lactaldehyde derived alkynols, which yields the glycal of amicetose or its epimer rhodinose, respectively [33]. An approach to amicetose (and a few other 6-desoxy sugars) involves the formation of an enantiopure β,γ-unsaturated δ-valerolactone via ring closing metathesis (RCM). The RCM product is subsequently converted into L-amicetose in four steps [34]. Unfortunately, the steps determining the configuration at C4 proceed in both cases with virtually no diastereoselectivity, although the resulting epimers were conveniently separated by chromatography. We have recently established two different metathesis based routes to L-amicetose [35] or L-amicetal [36], respectively, which both use enantiomerically pure L-ethyl lactate (**1**) as the starting material (Scheme 1). The synthetic routes rely on the highly diastereoselective two-step conversion of ethyl lactate to allylic alcohol **2** [36–37] and further to the RCM precursor **3**, which then undergoes RCM-isomerization to L-amicetal **4** [36]. Alternatively, we have used **2** as a substrate for a cross metathesis reaction with methyl acrylate under isomerization-free conditions to furnish enoate **5**, which underwent cyclization to the lactone **6** after hydrogenation. Reduction of **6** with DIBAL-H eventually yields a protected L-amicetose **7** [35]. To avoid the last step of the sequence, we envisaged a cross metathesis with acroleine, rather than methyl acrylate, to give enal **8** which should undergo spontaneous lactol formation after hydrogenation and desilylation. However, compared to cross metathesis reactions with methyl acrylate literature precedence for the use of acroleins as CM partner is significantly smaller [38–44].

In this contribution we report a de novo synthesis of a protected L-amicetose, using a cross metathesis reaction of allyl alcohol **2** with acrolein, and the elaboration of the cross metathesis product **8** to a fully protected acyclic cinerulose derivative. We are aware of only one previous de novo synthesis of DL-cinerulose, which used an Achmatowicz rearrangement of 2-(1-hydroxyethyl)furan [45].

## Results and Discussion

The three second generation catalysts **A** [46], **B** [47–48] and **C** [49–50] were initially tested for the cross metathesis of **2** and acrolein (Table 1). We started with the most common catalyst, the second generation Grubbs’ catalyst. In order to suppress undesired double bond isomerization reactions [51–52] we tried to avoid high reaction temperatures, which may cause catalyst decomposition to isomerization active species. This phenomenon has often been observed for second generation catalysts [53]. For these reasons, phenol was used as a rate accelerating additive. Phenols are believed to coordinate to the Ru and stabilize the catalytically active 14-electron species, resulting in a retarded catalyst decomposition [54–55]. In the presence of 5 mol % of **A** and 0.5 equiv of phenol, however, the yield of cross metathesis product **8** was very low at ambient temperature (Table 1, entry 1). Heating the mixture to reflux in dichloromethane resulted in a comparably low yield (Table 1, entry 2), whereas a significant improvement could be observed in toluene at 80 °C (Table 1, entry 3). We thought that the yield could not be further increased with catalyst **A** and therefore decided to test the phosphine free catalyst **B**. This and related catalysts [56], comprising a hemilabile *ortho*-isopropoxy substituted alkylidene ligand, are particularly suited for cross metathesis reactions [57–58]. Indeed, even at ambient temperature and without any rate accelerating additive, a good yield of 86% was obtained (Table 1, entry 4), which could be improved to quantitative by increasing the reaction temperature to 40 °C (Table 1, entry 5). Reducing the catalyst loading to 2.5 mol % is possible, but at the expense of isolated yield of product (Table 1, entry 6). We have previously used the Umicore M5_1_ catalyst (**C**) [49] for cross metathesis reactions and discovered that high selectivities and rates of conversion can be accomplished even with comparatively low catalyst loadings [59–60]. For the cross metathesis of **2** and acroleine, **C** gave significantly better yields than **A**, but was found to be inferior to **B** (Table 1, entries 7–9).

**Table 1:** Catalyst screening for CM of allyl alcohol **2** and acrolein.

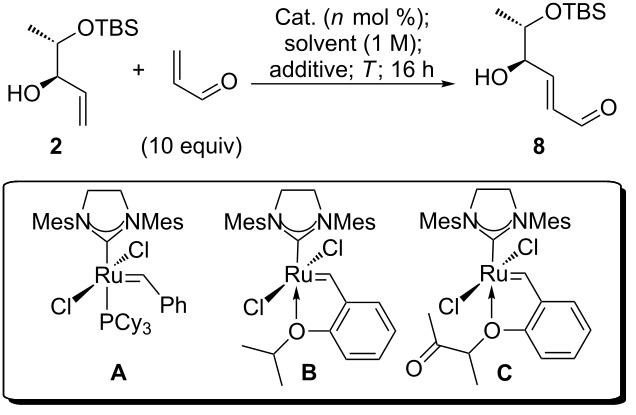						

entry	catalyst	cat. loading	additive (equiv)	solvent	*T*/°C	yield of **8**

1^a^	**A**	5 mol %	phenol (0.5)	CH_2_Cl_2_	25 °C	25%
2^a^	**A**	5 mol %	phenol (0.5)	CH_2_Cl_2_	40 °C	20%
3	**A**	5 mol %	phenol (0.5)	toluene	80 °C	68%
4	**B**	5 mol %	–	CH_2_Cl_2_	25 °C	86%
5	**B**	5 mol %	–	CH_2_Cl_2_	40 °C	99%
6	**B**	2.5 mol %	–	CH_2_Cl_2_	40 °C	76%
7	**C**	5 mol %	–	CH_2_Cl_2_	40 °C	65%
8	**C**	5 mol %	–	toluene	80 °C	80%
9	**C**	2.5 mol %	–	toluene	80 °C	65%

^a^Allylic alcohol **2** was recovered in 21% yield (entry 1) and in 12% yield (entry 2), respectively.

In the following step we decided to protect the hydroxy group at C4 first to avoid any complications arising from the formation of a furanose after hydrogenation of the C–C double bond. Benzoyl was chosen as a protecting group, because of its UV-activity, its orthogonality to the TBS group at C5–OH and its stability under hydrogenation conditions. Interestingly, attempted benzoylation in pyridine resulted exclusively in the formation of furan **10** (Table 2, entry 1). We reasoned that pyridine initiates an *E*/*Z*-isomerization of the enal through nucleophilic attack at the β-position, followed by lactol formation, benzoylation of the lactol and finally elimination of benzoic acid. Replacing pyridine as a solvent by dichloromethane and using NEt(iPr)_2_ as an HCl-scavenger led indeed to a suppression of the formation of furan **10**, but the desired benzoate **9** was obtained in yields lower than 20%, along with ca. 80% of recovered starting material **8** (Table 2, entry 2). Benzoate **9** was eventually obtained in high yields from benzoic acid using Steglich’s esterification [61] (Table 2, entry 3).

**Table 2:** C4–OH protection as benzoate.

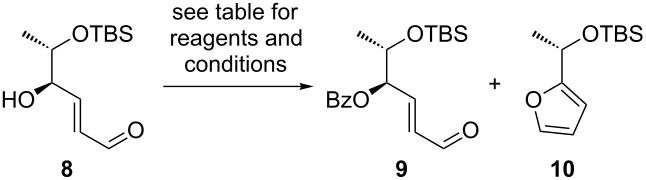			

entry	reagents and conditions	product	yield

1	benzoyl chloride (1.5 equiv), pyridine (0.4 M), 65 °C, 12 h	**10**	47%
2^a^	benzoyl chloride (2.6 equiv), NEt(iPr)_2_ (3.0 equiv), CH_2_Cl_2_, 40 °C, 12 h	**9**	<20%
3	benzoic acid (1.8 equiv), DCC (1.8 equiv), DMAP (0.1 equiv), CH_2_Cl_2_, 20 °C, 12 h	**9**	82%

^a^Starting material **8** was recovered in ca. 80% yield.

With enal **9** in hands, selective hydrogenation of the C–C double bond had to be accomplished in the next step. Lipshutz’ modification [62] of Stryker’s reagent [63], which we had previously used successfully for the conjugate reduction of a related enoate [35], failed completely in this case and resulted only in the isolation of unreacted starting material. For these reasons we resumed to a hydrogenation catalyzed by Pd/C, in spite of the well-known capricious nature of these transformations [64].

With a commercial sample of Pd on charcoal (10 wt %) a plethora of products was detected, four of which could be isolated and identified as the hydrogenated acetal **11**, the methyl ether **13**, and the two desilylated products **14** and **15** (Table 3, entry 1). Literature precedence exists for the Pd/C-induced dealkoxylation of acetals [65] and for desilylation reactions of silyl ethers [66]. With in situ generated Pd/C (obtained from Pd(OAc)_2_ and charcoal according to Felpin’s protocol) [67] an improved selectivity was observed as the cleavage of the silyl groups could be suppressed. However, reductive dealkoxylation of the acetal remained a problem and the combined yield of the desired products **11** and **12** was still unsatisfactory (Table 3, entry 2). This situation changed when we used Pd(OH)_2_ on charcoal (10 wt %) as hydrogenation catalyst, which resulted in the exclusive formation of acetal **11** and aldehyde **12** in 83% combined yield (Table 3, entry 3).

**Table 3:** Hydrogenation of benzoyl protected enal **9**.

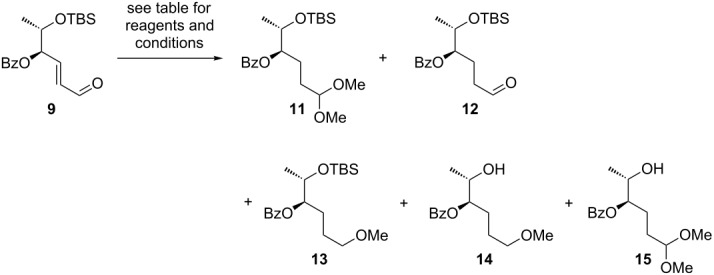						

entry	reagents and conditions	**11**	**12**	**13**	**14**	**15**

1	Pd/C (10 wt %; 1.6 mol %), H_2_ (1 bar), methanol, 20 °C, 12 h	31%	–	16%	16%	8%
2	Pd(OAc)_2_ (1 mol %), activated charcoal (9 mol %), H_2_ (1 bar), methanol, 20 °C, 12 h	28%	9%	trace	–	–
3^a^	Pd(OH)_2_/C (10 wt %; 1.2 mol %), H_2_ (1 bar), methanol, 20 °C, 12 h	51%	32%	–	–	–

^a^Products **11** and **12** were obtained as an inseparable mixture, yields are estimated from ^1^H NMR spectrum.

**Scheme 2:**
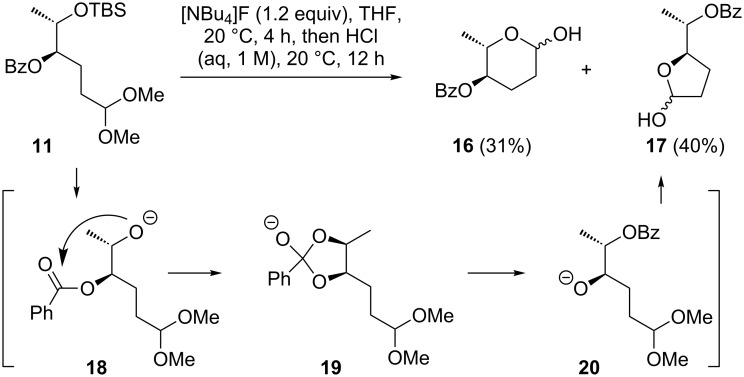
Two step desilylation–acetal hydrolysis.

For the deprotection of **11** and **12** a desilylation initiated by TBAF, followed by acidic hydrolysis was investigated first. With isolated acetal **11**, these conditions induced a scrambling of the benzoate and led to a mixture of the desired pyranose **16** and the furanose **17**. Facile migration of carboxylates upon TBAF-mediated desilylation of a vicinal alcohol has previously been observed by us in a different context [68]. We assume that this process starts with a nucleophilic attack of the alkoxylate **18** at the ester carbon, giving a five membered intermediate **19** (Scheme 2).

**Scheme 3:**
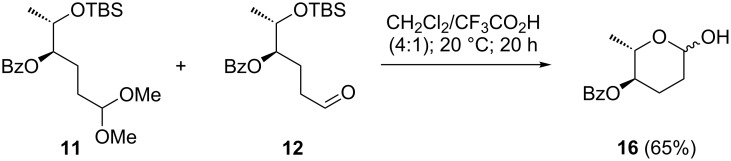
Deprotection of **11** and **12** to L-amicetose derivative **16**.

The benzoate scrambling was completely suppressed by avoiding highly nucleophilic alkoxide intermediates, which was achieved with acidic deprotection conditions. Thus, treating the mixture of **11** and **12** with trifluoroacetic acid in dichloromethane at ambient temperature resulted in desilylation and acetal cleavage, giving 4-benzoyl protected L-amicetose **16** as an anomeric mixture in 65% yield (Scheme 3).

**Scheme 4:**
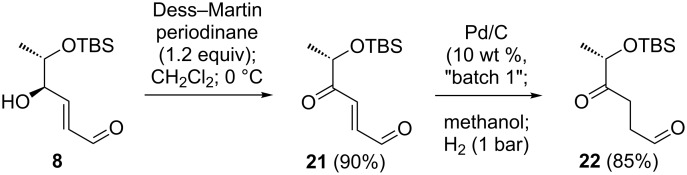
Synthesis of a cinerulose-TBS ether **22**.

With a view towards L-cinerulose, formally the C4-oxidation product of L-amicetose, we started from cross metathesis product **8** which was first oxidized using Dess–Martin periodinane [69]. The α,β-unsaturated γ-keto aldehyde **21** was obtained in a very high yield of 90%, and subsequently subjected to hydrogenation conditions using a commercial sample of Pd/C (10 wt %, “batch 1”). We were pleased to find that the expected cinerulose derivative **22** could be isolated in analytically pure form in 85% yield (Scheme 4).

To our great dismay, we found that this result could not be reproduced with a different batch of commercial Pd/C (10 wt %, “batch 2”), purchased from the same supplier. As can be seen from Table 4, entry 1, the second batch of Pd/C catalyzes under otherwise identical conditions a chemoselective acetalization of the aldehyde, leading to **23** which was isolated in 52% yield. A similar result was obtained with Pd(OH)_2_/C (Table 4, entry 2). In situ formed Pd on charcoal [67], prepared from 0.5 mol % of Pd(OAc)_2_ as a precursor, gave an inseparable mixture of starting material **21** and acetal **23** in a 1.7:1 ratio (Table 4, entry 3). By increasing the amount of catalyst precursor to 1 mol %, conversion to acetal **23** was complete, and this product could be isolated in 82% yield (Table 4, entry 4).

**Table 4:** Chemoselective acetalization of oxidation product **21**.

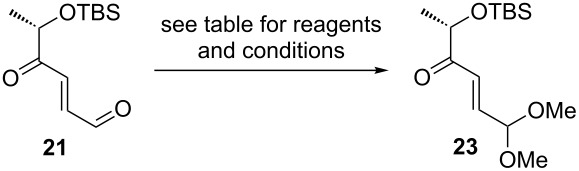		

entry	reagents and conditions	yield of **23**

1	Pd/C (“batch 2”, 10 wt %; 2.2 mol %), H_2_ (1 bar), methanol, 20 °C, 12 h	52%
2	Pd(OH)_2_/C (10 wt %; 1,7 mol %), H_2_ (1 bar), methanol, 20 °C, 12 h	43%
3	Pd(OAc)_2_ (0.5 mol %), activated charcoal (9 mol %), H_2_ (1 bar), methanol, 20 °C, 12 h	34%^a^
4	Pd(OAc)_2_ (1.0 mol %), activated charcoal (9 mol %), H_2_ (1 bar), methanol, 20 °C, 12 h	82%

^a^Estimated from ^1^H NMR spectrum (**21** and **23** were obtained as an inseparable mixture in ca. 90% combined yield).

Although the Pd(II)-catalyzed acetalization of aldehydes, including enals, is known [70], we are not aware of examples where Pd-catalysts immobilized on charcoal have been used for this transformation. Most likely the acetalization is catalyzed by residual Pd^2+^ via Lewis-acidic carbonyl activation. It is known that the reduction to Pd(0) is almost never complete, even if hydrogen is present [71–72].

At this stage we decided to reinvestigate the hydrogenation step, starting from acetal **23**, using fresh samples of catalyst which have not been used previously for the acetalization (Table 5). With in situ prepared Pd/C (Table 5, entry 1) a major amount of starting material **23** was recovered and minor quantities of hydrogenated products **24** and **26**, as well as desilylated starting material **25** were detected. With commercial Pd/C (10 wt %, “batch 2”) (Table 5, entry 2) the starting material was fully consumed and only hydrogenated products **24** and **26** could be detected in a 1:2 ratio, but unfortunately the isolated yields were only mediocre. As for the hydrogenation of benzoate **9** (Table 3), best results were reproducibly obtained with Pd(OH)_2_ on charcoal (Table 5, entry 3), leading to an isolated yield of 84% of a double protected cinerulose derivative **24**.

**Table 5:** Hydrogenation of acetal **23**.

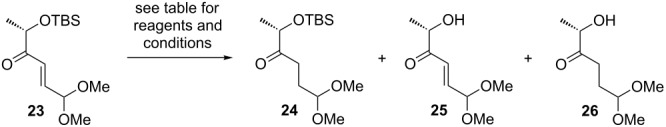				

entry	reagents and conditions	**24**	**25**	**26**

1^a^	Pd(OAc)_2_ (1.0 mol %), activated charcoal (9 mol %), H_2_ (1 bar), methanol, 20 °C, 12 h	7%	16%	7%
2	Pd/C (10 wt %; 2.2 mol %), H_2_ (1 bar), methanol, 20 °C, 12 h	19%	n.d.	38%
3	Pd(OH)_2_/C (10 wt %; 1.0 mol %), H_2_ (1 bar), methanol, 20 °C, 12 h	84%	n.d.	n.d.

^a^Starting material **23** (49%) was recovered.

**Scheme 5:**
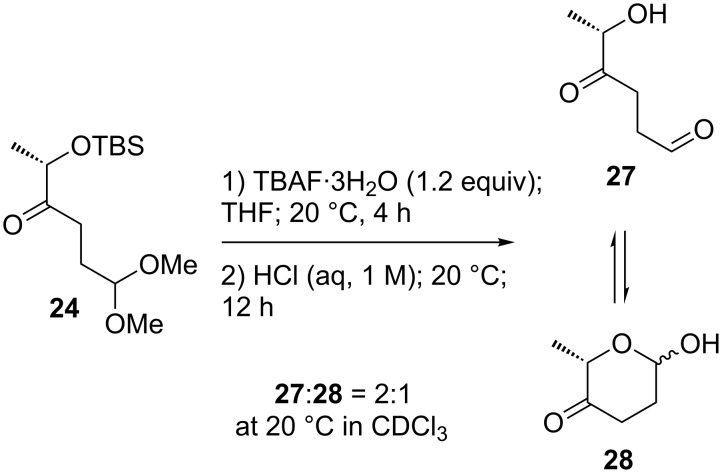
Deprotection of **24**.

All attempts to isolate cinerulose were hampered by the high volatility of the product. To accomplish complete deprotection of the precursor, compound **24** was first treated with TBAF trihydrate in THF, followed by treatment with diluted aqueous HCl. Although only small amounts of material were obtained via this procedure, ^1^H NMR spectroscopical analysis was possible. The data suggest that cinerulose exists in CDCl_3_ as a 2:1 mixture of acyclic aldose **27** and lactol **28** (Scheme 5).

## Conclusion

In summary, we showed that an allylic alcohol, available diastereoselectively from enantiomerically pure L-lactate in few steps, is a useful starting material for a metathesis based synthesis of protected L-amicetose and L-cinerulose. It undergoes a clean and high yielding cross metathesis reaction with acrolein, however, the most commonly used second generation Grubbs’ catalyst is not the ideal initiator for this particular transformation. Significantly better results were obtained with two different phosphine free catalysts comprising a hemilabile alkoxy substituted benzylidene ligand, even at only moderately elevated temperatures. The acrolein cross metathesis product can be converted into the 4-benzoate of L-amicetose via benzoylation, Pd-catalyzed hydrogenation and global deprotection, whereas a cinerulose derivative protected at C1 as a dimethyl acetal and at C5–OH as a TBS ether becomes available via Dess–Martin oxidation, Pd-catalyzed acetalization and Pd-catalyzed hydrogenation. Notably, our results underline once again that apparently trivial olefin hydrogenation reactions catalyzed by commercial “Pd/C” can be capricious and very difficult to reproduce, unless the specific properties of the catalyst used and the conditions for its preparation are documented by the supplier. Unfortunately, this is very often not the case and the catalytic activities of apparently identical catalysts which are often designated just as “10 wt % Pd on carbon” may vary dramatically.

## Supporting Information

File 1 Experimental procedures and analytical data.

File 2 Copies of ^1^H and ^13^C NMR spectra.
