# Supplementary material for: Olefin cross metathesis based de novo synthesis of a partially protected L-amicetose and a fully protected L-cinerulose derivative
Source: Beilstein J Org Chem. 2014 May 6;10:1023–31. doi: 10.3762/bjoc.10.102 (PMC4077472; doi:10.3762/bjoc.10.102)
Supplement: File 1 — Experimental procedures and analytical data. [file Beilstein_J_Org_Chem-10-1023-s001.pdf]

Supporting Information File 1

for

Olefin cross metathesis based de novo synthesis of a partially protected L-amicetose and a fully protected L-cinerulose derivative

Bernd Schmidt* and Sylvia Hauke

Address: Institut für Chemie, Organische Synthesechemie, Universität Potsdam, Karl-Liebknecht-Straße 24–25, 14476 Potsdam-Golm, Germany

Email: Bernd Schmidt - bernd.schmidt@uni-potsdam.de

* Corresponding author

Experimental procedures and analytical data

Experimental

General experimental methods. All experiments were conducted in dry reaction vessels under an atmosphere of dry nitrogen. Solvents were purified using a commercial solvent purification system. ^{1}H NMR spectra were obtained at 300 MHz or at 500 MHz in $CDCl_3$ with $CHCl_3$ (δ = 7.26 ppm) as an internal standard. Coupling constants (*J*) are given in Hz. ^{13}C NMR spectra were recorded at 125 MHz or at 75 MHz in $CDCl_3$ with $CDCl_3$ (δ = 77.0 ppm) as an internal standard. The number of coupled protons was analyzed by APT-experiments and is denoted by a number in parantheses following the chemical shift value. IR spectra were recorded with a FTIR-spectrometer with an ATR-crystal unit. Wavenumbers (ν) are given in cm^{-1}. The peak intensities are defined as strong (s), medium (m) or weak (w). Mass spectra were obtained either by EI or ESI/TOF measurements. Alcohol **2** was synthesized following a previously published procedure [1].

(4*R*,5*S*,*E*)-5-(*tert*-Butyldimethylsilyloxy)-4-hydroxyhex-2-enal (8). To a solution of **2** (500 mg, 2.3 mmol) and acroleine (1.7 mL, 23.1 mmol) in dry dichloromethane (2.3 mL, 1.0 M) was added Ru-catalyst **B** (97.6 mg, 0.12 mmol, 5 mol %). The mixture was heated to 40 °C and stirred for 16 h at this temperature. After removing all volatiles in vacuo the residue was purified by column chromatography on silica to give **8** (560 mg, 2.3 mmol, 99%). $[\alpha]^{D}_{23}$ = +33.0 (*c* 0.48, CH_2Cl_2); ^{1}H NMR (500 MHz, $CDCl_3$) δ 9.59 (d, *J* = 7.9, 1H), 6.78 (dd, *J* = 15.8, 4.7, 1H), 6.36 (ddd, *J* = 15.7, 7.9, 1.6, 1H), 4.33 (m, 1H), 3.97 (qd, *J* = 6.3, 4.2, 1H), 2.51 (bs, 1H), 1.11 (d, *J* = 6.3), 0.90 (s, 9H), 0.10 (s, 6H); ^{13}C NMR (125 MHz, $CDCl_3$) δ 193.3 (0), 154.4 (1), 132.3 (1), 75.0 (1), 70.7 (1), 25.7 (3), 20.1 (0), 18.1 (3), –4.4 (3), –4.9 (3); IR (ATR) ν 2930 (w), 1691 (m), 1253 (m), 1089 (m), 831 (s), 775 (s); MS (EI) *m/z* 227 (6), 187 (14),

169 (15), 159 (100), 145 (16), 115 (19), 73 (58), 43 (50); HRMS (ESI) calcd for $C_{12}H_{24}O_3NaSi$ $[M+Na]^+$ 267.1392, found 267.1388. Anal. calcd for $C_{12}H_{24}O_3Si$: C, 59.0; H, 9.9. Found: C, 59.1; H, 9.9.

(2*S*,3*R*,*E*)-2-(*tert*-Butyldimethylsilyloxy)-6-oxohex-4-en-3-yl benzoate (9). A solution of **8** (738 mg, 3.0 mmol), benzoic acid (443 mg, 3.6 mmol), dicyclohexyl-carbodiimide (746 mg, 3.6 mmol) and DMAP (37 mg, 0.3 mmol) in dry dichloromethane (8.2 mL) was stirred at 20 °C for 12 h. The mixture was filtered and the solid was washed several times with MTBE. The combined organic layers were washed with HCl (aq, 1 M), followed by a saturated aqueous solution of $NaHCO_3$. The organic extracts were dried with $MgSO_4$, filtered and evaporated. The residue was purified by column chromatography to give **9** (860 mg, 1.6 mmol, 82%) as a colourless liquid. $[\alpha]^{25}_D$ = +64.9 (*c* 0.59, CH_2Cl_2); ^{1}H NMR (300 MHz, $CDCl_3$) δ 9.60 (d, *J* = 7.8, 1H), 8.07 (dm, *J* = 7.1, 1H), 7.59 (m, 1H), 7.53-7.42 (m, 2H), 6.97 (dd, *J* = 15.9, 5.1, 1H), 6.32 (ddd, *J* = 15.9, 7.8, 1.5, 1H), 5.60 (ddd, *J* = 5.1, 4.7, 1.5, 1H), 4.16 (qd, *J* = 6.3, 4.7, 1H), 1.27 (d, *J* = 6.3, 1H), 0.88 (s, 9H), 0.06 (s, 3H), 0.05 (s, 3H); ^{13}C NMR (75 MHz, $CDCl_3$) δ 192.9, 165.4, 151.1, 133.4, 133.1, 129.7, 129.5, 128.5, 76.9, 69.8, 25.6, 20.4, 17.9, −4.5, −4.9; IR (ATR) ν 2930 (m), 1697 (s), 1108 (s), 1040 (s), 834 (s); MS (EI) *m/z* 304 (7), 247 (10), 179 (94), 159 (38), 105 (100), 73 (22); HRMS (ESI) calcd for $C_{19}H_{29}O_4Si$ $[M+H]^+$ 349.1835, found 349.1806. Anal. calcd for $C_{19}H_{28}O_4Si$: C, 65.5; H, 8.1. Found: C, 65.1; H, 7.6.

(*S*)-*tert*-Butyl(1-(furan-2-yl)ethoxy)dimethylsilane (10). A solution of **8** (250 mg, 1.0 mmol) and benzoyl chloride (0.176 mL, 1.5 mmol) in pyridine (25 mL) was stirred at 65 °C for 12 h. After cooling to ambient temperature, the reaction mixture was poured onto ice water, and the aqueous layer was extracted three times with MTBE.

The combined organic layers were washed with a saturated aqueous solution of $NaHCO_3$ and dried with $MgSO_4$. After filtration, the solvent was removed under reduced pressure and the residue purified by chromatography on silica to furnish **10** (107 mg, 0.5 mmol, 47%) as a colourless liquid. $[\alpha]^{25}_{D}$ –23.6 (*c* 0.57, CH_2Cl_2); ^1H NMR (300 MHz, $CDCl_3$) δ 7.33 (dd, *J* = 1.8, 0.8, 1H), 6.30 (dd, *J* = 3.2, 1.8, 1H), 6.16 (ddd, *J* = 3.2, 0.8, 0.8, 1H), 4.88 (q, *J* = 6.4, 1H), 1.48 (d, *J* = 6.5, 3H), 0.90 (s, 9H), 0.09 (s, 3H), 0.03 (s, 3H); ^{13}C NMR (75 MHz, $CDCl_3$) δ 158.3, 141.2, 109.9, 104.7, 64.5, 25.8, 22.9, 18.2, –4.9, –5.0; IR (ATR) ν 2930 (m), 1256 (m), 1152 (m), 1094 (m), 830 (s), 775 (s), 731 (s); MS (EI) *m/z* 211 (2), 169 (50), 95 (21), 75 (100), 57 (19); HRMS (ESI) calcd for $C_{12}H_{23}O_2Si$ $[M+H]^+$ 227.1467, found 251.1471. Anal. calcd for $C_{12}H_{22}O_2Si$: C, 63.7; H, 9.8. Found: C, 63.4; H, 9.7.

Hydrogenation of enal 9 with Pd/C. To a solution of **9** (250 mg, 0.72 mmol) in dry and degassed methanol (2 mL) was added Pd/C (10 wt %, 12.5 mg, 1.6 mol %). The suspension was stirred for 12 h under an atmosphere of hydrogen (1 bar). The mixture was filtered through a pad of celite, which was subsequently washed three times with MTBE. After evaporation of all volatiles the residue was chromatographed on silica to furnish **11** (89 mg, 0.22 mmol, 31%) as a colourless liquid, **13** (42 mg, 0.11 mmol, 16%) as a colourless liquid, and a fraction containing the inseparable alcohols **14** and **15** (ca. 45 mg, ca. 0.17 mmol, ca. 24% combined yield) in a 2:1 ratio (^1H NMR). Analytical data for (2*S*,3*R*)-2-(*tert*-butyldimethylsilyloxy)-6,6-dimethoxyhexan-3-yl benzoate (**11**): $[\alpha]^{24}_{D}$ +19.8 (*c* 0.33, CH_2Cl_2); ^1H NMR (300 MHz, $CDCl_3$) δ 8.08-8.00 (m, 2H), 7.55 (m, 1H), 7.50-7.38 (m, 2H), 5.00 (td, *J* = 8.0, 4.2, 1H), 4.38 (t, *J* = 5.6, 1H), 4.02 (qd, *J* = 6.3, 4.2, 1H), 3.30 (s, 6H), 1.86-1.53 (m, 4H), 1.19 (d, *J* = 6.3, 3H) 0.87 (s, 9H), 0.02 (s, 3H), 0.00 (s, 3H); ^{13}C NMR (75 MHz,

$CDCl_3$) δ 166.2, 132.8, 130.5, 129.6, 128.3, 104.3, 78.2, 69.5, 52.9, 52.6, 28.7, 25.7, 24.0, 19.8, 18.0, −4.5, −4.9; IR (ATR) ν 2930 (m), 1717 (s), 1281 (s), 1115 (s), 835 (s), 775 (s), 710 (s); HRMS (ESI) calcd for $C_{21}H_{36}O_5NaSi$ $[M+Na]^+$ 419.2230, found 419.2219. Analytical data for (2*S*,3*R*)-2-(*tert*-butyldimethylsilyloxy)-6-methoxyhexan-3-yl benzoate (**13**): ^1H NMR (300 MHz, $CDCl_3$) δ 8.08-8.15 (m, 2H), 7.55 (m, 1H), 7.48-7.38 (m, 2H), 5.02 (td, *J* = 6.0, 4.1, 1H), 4.03 (qd, *J* = 6.3, 4.1, 1H), 3.39 (td, *J* = 6.5, 1.4, 2H), 3.31 (s, 3H), 1.86-1.52 (m, 4H), 1.19 (d, *J* = 6.3, 3H) 0.87 (s, 9H), 0.02 (s, 3H), 0.00 (s, 3H); ^{13}C NMR (75 MHz, $CDCl_3$) δ 166.3 (0), 132.8 (1), 130.6 (0), 129.6 (1), 128.3 (1), 78.3 (1), 72.4 (2), 69.5 (1), 58.5 (3), 25.8 (2), 25.7 (3), 25.6 (2), 19.8 (3), 18.0 (0), −4.5 (3), −4.9 (3); IR (ATR) ν 2929 (m), 1717 (s), 1271 (s), 1115 (s), 835 (s), 775 (s), 709 (s).

Hydrogenation of enal 9 with $Pd(OH)_2$/C. To a solution of **8** (250 mg, 0.72 mmol) in dry and degassed methanol (2 mL) was added $Pd(OH)_2$/C (10 wt %, 12.5 mg, 1.2 mol %). The suspension was stirred for 12 h under an atmosphere of hydrogen (1 bar). The mixture was filtered through a pad of celite, and the pad was washed three times with MTBE. Evaporation of the solvents furnished an inseparable mixture of **11** and **12** (226 mg, ca. 0.61 mmol, ca. 83% combined yield). Selected analytical data for (2*S*,3*R*)-2-(*tert*-butyldimethylsilyloxy)-6-oxohexan-3-yl benzoate (**12**) obtained from the mixture: ^1H NMR (300 MHz, $CDCl_3$) δ ^1H NMR (300 MHz, $CDCl_3$) 9.78 (t, *J* = 1.2, 1H), 8.10-8.00 (2H), 7.61-7.51 (1H), 7.49-7.38 (2H), 4.99 (m, 1H), 4.04 (m, 1H), 2.56 (td, *J* = 7.5, 1.0, 2H), 2.15-2.07 (2H), 1.21 (d, *J* = 6.3, 3H) 0.88 (s, 9H), 0.03 (s, 3H), 0.00 (s, 3H).

L-Amicetose-4-benzoate (16). The crude mixture of **11** and **12** (149 mg, ca. 0.38 mmol), obtained from the $Pd(OH)_2$/C-catalyzed hydrogenation as described above, was dissolved in dry dichloromethane (1.5 mL) and CF_3CO_2H (0.4 mL) was added at ambient temperature. The solution was stirred at ambient temperature for 20 h, followed by evaporation of all volatiles. The residue was redissolved in MTBE, and the solution was washed with a saturated aqueous solution of $NaHCO_3$. The organic layer was separated and dried with $MgSO_4$, filtered and evaporated. The residue was purified by chromatography on silica to furnish **16** (58 mg, 0.24 mmol, 65%) as a 58:42 mixture of anomers. $[\alpha]^{25}_{D}$ −53.4 (*c* 0.65, CH_2Cl_2); ^{1}H NMR (300 MHz, $CDCl_3$) δ 8.03 (ddm, *J* = 7.1, 6.5, 2H), 7.57 (dd, *J* = 7.2, 7.0, 1H), 7.44 (dd, *J* = 7.7, 7.4, 2H), 5.30 (m, 0.5H, major), 4.89 (dm, *J* = 6.5, 0.5H, minor), 4.81-4.65 (m, 1H), 4.23 (m, 0.5H, major), 3.75 (m, 0.5H, minor), 3.27 (bs, 0.5H, minor) 2.84 (bs, 0.5H, major), 2.36-1.96 (m, 2H), 1.96-1.56 (m, 2H), 1.30 (d, *J* = 6.2, 1.5H), 1.22 (d, *J* = 6.3, 1.5H); ^{13}C NMR (75 MHz, $CDCl_3$) δ 165.8, 133.1, 133.0, 130.2, 130.1, 129.6, 129.6, 95.8, 90.9, 74.0, 73.6, 73.2, 66.8, 31.8, 29.2, 27.5, 23.5, 18.2, 18.0; IR (ATR) ν 3424 (m), 2937 (w), 1718 (s), 1316 (m), 1269 (s), 1114 (m), 1069 (m), 998 (m), 711 (s); HRMS (ESI) calcd for $C_{13}H_{15}O_3$ $[M–OH]^+$ 219.1021, found 219.1010.

Stepwise deprotection of 11. A solution of **11** (265 mg, 0.70 mmol) and TBAF·$3H_2O$ (253 mg, 0.80 mmol) in THF (6.7 mL) was stirred for 4 h at ambient temperature. Aqueous HCl (1 M) was then added and stirring was continued for 12 h at ambient temperature. A saturated aqueous solution of $NaHCO_3$ and MTBE were then added, and the organic layer was separated. The aqueous layer was extracted twice with MTBE, the combined organic extracts were dried with $MgSO_4$, filtered and evaporated. The residue was chromatographed on silica to give a 1:1.7 mixture of lactols **16** and **17** (112 mg, 0.50 mmol, 71% combined yield). By repeated careful

chromatography a sample of the furanose product (*S*)-1-((*R*)-5-hydroxytetrahydrofuran-2-yl)ethyl benzoate (**17**) could be isolated as a 54 : 46 mixture of anomers for analytical purposes: $[\alpha]^{25}_{D}$ +12.0 (*c* 0.80, CH_2Cl_2); ^{1}H NMR (300 MHz, $CDCl_3$) δ 8.10-7.96 (m, 2H), 7.61-7.49 (m, 1H), 7.49-7.36 (m, 2H), 5.57 (d, *J* = 3.9, 0.5H, minor), 5.51 (d, *J* = 4.0, 0.5H, major), 5.35-5.05 (m, 1H), 4.36 (m, 0.5H, minor), 4.18 (m, 0.5H, major), 3.35 (bs, 1H) 2.33-1.46 (m, 4H), 1.38 (d, *J* = 6.3, 1.5H), 1.33 (d, *J* = 6.4, 1.5H); ^{13}C NMR (75MHz, $CDCl_3$) δ 165.9 (0), 132.9 (1), 129.6 (1), 129.6 (1), 128.3 (1), 99.0 (1), 98.6 (1), 82.6 (1), 80.2 (1), 72.3 (1), 72.0 (1), 33.6 (2), 32.6 (2), 24.8 (2), 24.7 (2), 16.4 (3), 16.2 (3); IR (ATR) ν 3423 (m), 2953 (w), 1713 (s), 1451 (m), 1268 (s), 1112 (m), 1069 (m), 988 (m), 709 (s); HRMS (ESI) calcd for $C_{13}H_{15}O_3$ $[M–OH]^+$ 219.1021, found 219.1016.

(*S*,*E*)-5-(*tert*-Butyldimethylsilyloxy)-4-oxohex-2-enal (21). To a solution of **8** (800 mg, 3.3 mmol) in dry dichloromethane (20 mL) was added Dess–Martin periodinane (1.67 g, 3.9 mmol) at 0 °C, and the mixture was stirred for 2 h at ambient temperature. The reaction mixture was diluted with MTBE, and subsequently washed with saturated aqueous solutions of $NaHCO_3$ and $Na_2S_2O_3$. The aqueous layer was extracted with MTBE, and the combined organic extracts were dried with $MgSO_4$, filtered and evaporated. The residue was chromatographed on silica to furnish **21** (712 mg, 2.9 mmol, 90%) as a yellowish liquid. $[\alpha]^{24}_{D}$ –10.1 (*c* 0.51, CH_2Cl_2); ^{1}H NMR (300 MHz, $CDCl_3$) δ 9.76 (d, *J* = 7.6, 1H), 7.42 (d, *J* = 16.0, 1H), 6.92 (dd, *J* = 16.0, 7.6, 1H), 4.36 (q, *J* = 6.8, 1H), 1.35 (d, *J* = 6.8, 3H), 0.90 (s, 9H), 0.10 (s, 3H), 0.07 (s, 3H); ^{13}C NMR (75 MHz, $CDCl_3$) δ 201.2 (0), 192.9 (1), 140.1 (1), 138.6 (1), 74.5 (1), 25.7 (3), 20.7 (3), 18.1 (0), −4.7 (3), −5.0 (3); IR (ATR) ν 2932 (w), 1696 (m), 1254 (m), 1112 (m), 832 (s), 777 (s); MS (EI) *m/z* 185 (77), 159 (68), 157 (69), 143

(13), 115 (26), 75 (62), 73 (50), 57 (21), 43 (25); HRMS (ESI) calcd for $C_{12}H_{23}O_3Si$ $[M+H]^+$ 243.1400, found 243.1386. Anal. calcd for $C_{12}H_{22}O_3Si$: C, 59.5; H, 9.2. Found: C, 59.3; H, 9.0.

(*S*)-5-(*tert*-Butyldimethylsilyloxy)-4-oxohexanal (22). To a solution of **21** (380 mg, 1.57 mmol) in dry and degassed methanol (50 mL) was added Pd/C ("batch 1", 10 wt %, 38 mg, 2.2 mol %). The suspension was stirred for 12 h under an atmosphere of hydrogen (1 bar). The mixture was filtered through a pad of Celite®, and the pad was washed three times with MTBE. Evaporation of the solvents furnished **22** (326 mg, 1.33 mmol, 85%) as a colourless liquid. $[\alpha]^{23}_D$ –3.6 (*c* 0.56, CH_2Cl_2); ^1H NMR (300 MHz, $CDCl_3$) δ 9.79 (s, 1H), 4.19 (q, *J* = 6.8, 1H), 2.97 (ddd, *J* = 19.0, 6.4, 6.4, 1H), 2.83 (ddd, *J* = 19.0, 6.7, 5.3, 1H), 2.76-2.68 (m, 2H), 1.30 (d, *J* = 6.8, 3H), 0.91 (s, 9H), 0.08 (s, 6H); ^{13}C NMR (75 MHz, $CDCl_3$) δ 213.1 (0), 200.3 (1), 74.8 (1), 37.1 (2), 29.6 (2), 25.7 (3), 20.9 (3), 18.0 (0), −4.7 (3), −5.1 (3); IR (ATR) *ν* 2932 (w), 1716 (m), 1117 (m), 832 (s), 776 (s); MS (EI) *m/z* 187 (31), 159 (69), 143 (100), 73 (67); HRMS (ESI) calcd for $C_{12}H_{24}O_3Si$ $[M]^+$ 244.1495, found 244.1486. Anal. calcd for $C_{12}H_{24}O_3Si$: C, 59.0; H, 9.9. Found: C, 58.7; H, 10.0.

(*S*,*E*)-2-(*tert*-Butyldimethylsilyloxy)-6,6-dimethoxyhex-4-en-3-on (23). Details for conditions listed in Table 4, entry 1: to a solution of **21** (357 mg, 1.50 mmol) in dry and degassed methanol (50 mL) was added Pd/C ("batch 2", 10 wt %, 36 mg, 2.2 mol %). The suspension was stirred for 12 h under an atmosphere of hydrogen (1 bar). The mixture was filtered through a pad of Celite®, and the pad was washed three times with MTBE. Evaporation of the solvents furnished **23** (218 mg, 0.80 mmol, 52%) as a colourless liquid.

Details for conditions listed in Table 4, entry 4: Enal **21** (1.10 g, 4.5 mmol) was dissolved in dry methanol (20 mL) and $Pd(OAc)_2$ (10.2 mg, 0.045 mmol, 1 mol %) and activated charcoal (92 mg) were added. The reaction vessel was flushed with hydrogen, and the mixture was stirred under an atmosphere of hydrogen (1 bar) for 12 h. The mixture was filtered through a pad of Celite®, and the pad was washed three times with MTBE. All volatiles were evaporated to furnish **23** (1.10 g, 3.70 mmol, 82%) as a colourless liquid. $[\alpha]^{23}_{D}$ –8.4 (*c* 0.62, CH_2Cl_2); ^1H NMR (300 MHz, $CDCl_3$) δ 6.87 (dd, *J* = 16.0, 1.0, 1H), 6.74 (dd, *J* = 16.0, 3.7, 1H), 4.95 (dd, *J* = 3.7, 1.0, 1H), 4.27 (q, *J* = 6.8, 1H), 3.32 (s, 3H), 3.32 (s, 3H), 1.31 (d, *J* = 6.8, 3H), 0.09 (s, 9H), 0.06 (s, 3H), 0.05 (s, 3H); ^{13}C NMR (75 MHz, $CDCl_3$) δ 201.5 (0), 141.4 (1), 126.7 (1), 101.1 (1), 74.5 (1), 52.8 (3), 52.8 (3), 25.7 (3), 20.8 (3), 18.2 (0), −4.8 (3), −5.0 (3); IR (ATR) ν 2933 (w), 1702 (m), 1256 (m), 1123 (s), 1056 (s), 832 (s), 777 (s); MS (EI) *m/z* 231 (32), 159 (32), 75 (100), 73 (98), 43 (22); HRMS (ESI) calcd for $C_{14}H_{28}O_4NaSi$ $[M+Na]^+$ 311.1655, found 311.1659. Anal. calcd for $C_{14}H_{28}O_4Si$: C, 58.3; H, 9.8. Found: C, 58.1; H, 9.7.

(*S*)-2-(*tert*-Butyldimethylsilyloxy)-6,6-dimethoxyhexan-3-on (24). To a solution of **23** (225 mg, 0.80 mmol) in dry and degassed methanol (20 mL) was added $Pd(OH)_2/C$ (10 wt %, 11 mg, 1.0 mol %). The suspension was stirred for 12 h under an atmosphere of hydrogen (1 bar). The mixture was filtered through a pad of Celite®, and the pad was washed three times with MTBE. All volatiles were evaporated and the residue was purified by chromatography on silica to furnish **24** (190 mg, 0.70 mmol, 84%). $[\alpha]^{24}_{D}$ –4.1 (*c* 0.23, CH_2Cl_2); ^1H NMR (300 MHz, $CDCl_3$) δ 4.36 (t, *J* = 5.7, 1H), 4.14 (q, *J* = 6.8, 1H), 3.30 (s, 6H), 2.65 (t, *J* = 7.1, 2H), 1.86 (td, *J* = 7.1, 5.8, 2H), 1.27 (d, *J* = 6.8, 3H), 0.91 (s, 9H), 0.07 (s, 3H), 0.07 (s, 3H); ^{13}C NMR (75 MHz, $CDCl_3$) δ 213.4 (0), 103.8 (1), 74.9 (1), 53.0 (3), 53.0 (3), 31.8 (2), 26.0 (2), 25.7 (3),

20.8 (3), 18.1 (0), −4.7 (3), −5.1 (3); IR (ATR) ν 2931 (w), 2856 (w), 1718 (m), 1118 (m), 834 (s), 777 (s); HRMS (ESI) calcd for $C_{13}H_{27}O_3Si$ $[M–OMe]^+$ 259.1729, found 259.1703.

Deprotection of 24. To a solution of **24** (51 mg, 0.18 mmol) in THF (1.8 mL) was added TBAF·$3H_2O$ (67 mg, 0.21 mmol). The mixture was stirred for 4 h at ambient temperature, followed by addition of aqueous HCl (1 M, 5.0 mL). Stirring at ambient temperature was continued for 12 h. The reaction mixture was then neutralized by the addition of a saturated aqueous solution of $NaHCO_3$, and the aqueous layer was extracted three times with MTBE. The solvents were carefully removed in vacuo, and the residue was purified by chromatography on silica to furnish L-cinerulose (5 mg, 0.04 mmol, ca. 20%) as a 2:1 mixture of its open chain aldose form **27** and its aldopyranose form **28**. Analytical data were obtained from the mixture: $[\alpha]^{22}_{D}$ –15.5 (*c* 0.25, $CDCl_3$); IR (ATR) ν 3425 (bm), 2938 (w), 1717 (s), 1369 (w), 1095 (s), 1012 (s); selected data for **27**: ^1H NMR (300 MHz, $CDCl_3$) δ 9.81 (s, 1H), 4.46 (q, J = 7.1, 1H), 1.43 (d, J = 7.1, 3H); selected data for **28**: ^1H NMR (300 MHz, $CDCl_3$) δ 5.45 (t, J = 4.9, 1H), 4.46 (q, J = 6.7, 1H), 1.29 (d, J = 6.7, 3H).

Reference

1. Schmidt, B.; Biernat, A. *Chem. Eur. J.* **2008**, *14*, 6135–6141.
