# Supplementary material for: Olefin cross metathesis based de novo synthesis of a partially protected L-amicetose and a fully protected L-cinerulose derivative
Source: Beilstein J Org Chem. 2014 May 6;10:1023–31. doi: 10.3762/bjoc.10.102 (PMC4077472; doi:10.3762/bjoc.10.102)

Supporting Information File 2

for

Olefin cross metathesis based de novo synthesis of a partially protected L-amicetose and a fully protected L-cinerulose derivative

Bernd Schmidt* and Sylvia Hauke

Address: Institut für Chemie, Organische Synthesechemie, Universität Potsdam, Karl-Liebknecht-Straße 24–25, 14476 Potsdam-Golm, Germany

Email: Bernd Schmidt - bernd.schmidt@uni-potsdam.de

* Corresponding author

Copies of ^{1}H and ^{13}C NMR spectra

Table of Contents

^{1}H NMR ($CDCl_3$, 500 MHz) of **8**	S3
^{13}C NMR ($CDCl_3$, 125 MHz) of **8**	S4
^{1}H NMR ($CDCl_3$, 300 MHz) of **9**	S5
^{13}C NMR ($CDCl_3$, 75 MHz) of **9**	S6
^{1}H NMR ($CDCl_3$, 300 MHz) of **10**	S7
^{13}C NMR ($CDCl_3$, 75 MHz) of **10**	S8
^{1}H NMR ($CDCl_3$, 300 MHz) of **11**	S9
^{13}C NMR ($CDCl_3$, 75 MHz) of **11**	S10
^{1}H NMR ($CDCl_3$, 300 MHz) for hydrogenation of enal **9** (inseparable mixture of **11** and **12**, see table 3, entry 3)	S11
^{1}H NMR ($CDCl_3$, 300 MHz) of **13**	S12
^{13}C NMR-APT ($CDCl_3$, 75 MHz) of **13**	S13
^{1}H NMR ($CDCl_3$, 300 MHz) of **16**	S14
^{13}C NMR ($CDCl_3$, 75 MHz) of **16**	S15
^{1}H NMR ($CDCl_3$, 300 MHz) of **17**	S16
^{13}C NMR-APT ($CDCl_3$, 75 MHz) of **17**	S17
^{1}H NMR ($CDCl_3$, 300 MHz) of **21**	S18
^{13}C NMR-APT ($CDCl_3$, 75 MHz) of **21**	S19
^{1}H NMR ($CDCl_3$, 300 MHz) of **22**	S20
^{13}C NMR-APT ($CDCl_3$, 75 MHz) of **22**	S21
^{1}H NMR ($CDCl_3$, 300 MHz) of **23**	S22
^{13}C NMR-APT ($CDCl_3$, 75 MHz) of **23**	S23
^{1}H NMR ($CDCl_3$, 300 MHz) of **24**	S24
^{13}C NMR ($CDCl_3$, 75 MHz) of **24**	S25
^{1}H NMR ($CDCl_3$, 300 MHz) for the mixture obtained from deprotection of **24**	S26

^{1}H NMR ($CDCl_3$, 500 MHz) of **8**

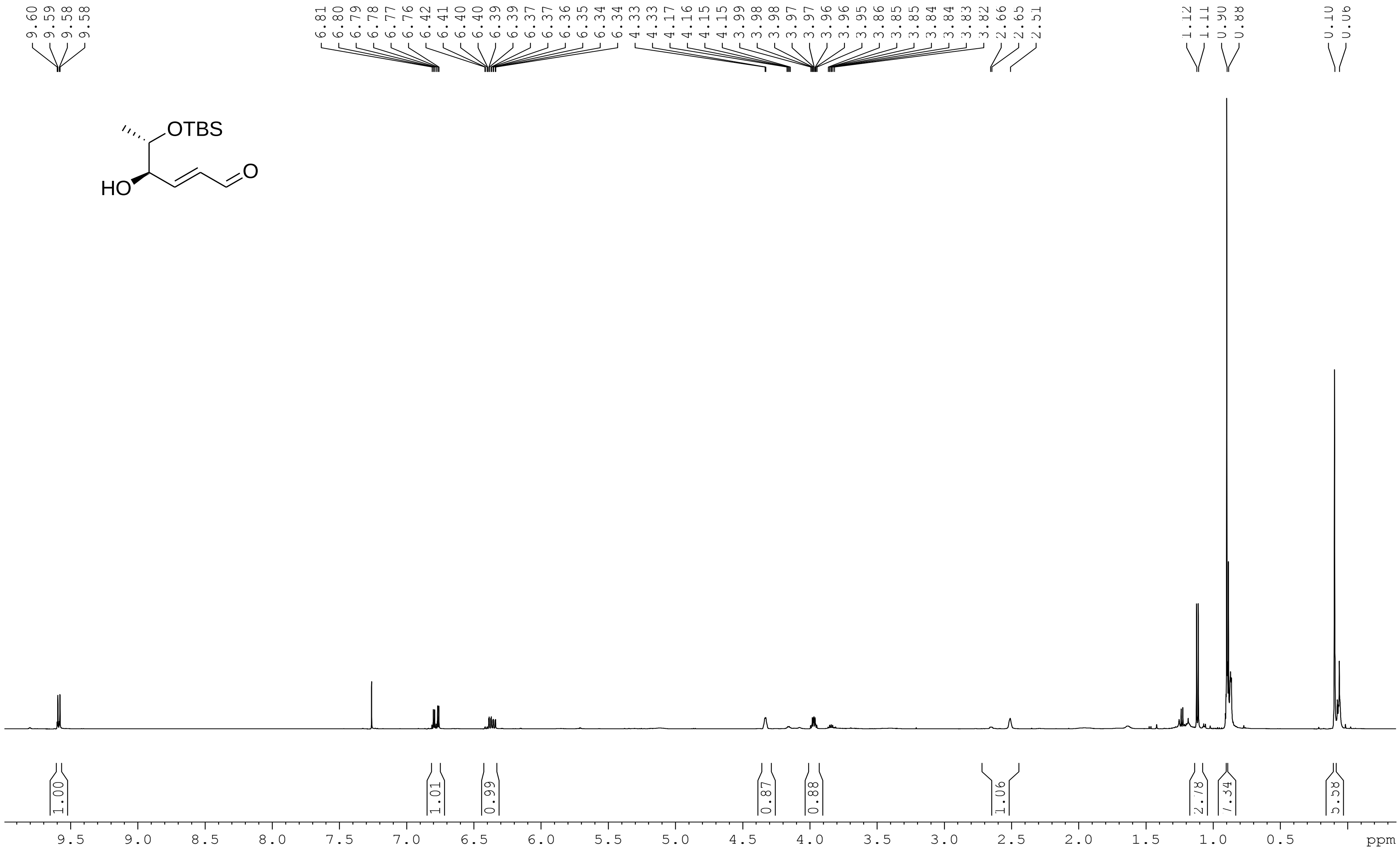

^{13}C NMR ($CDCl_3$, 125 MHz) of **8**

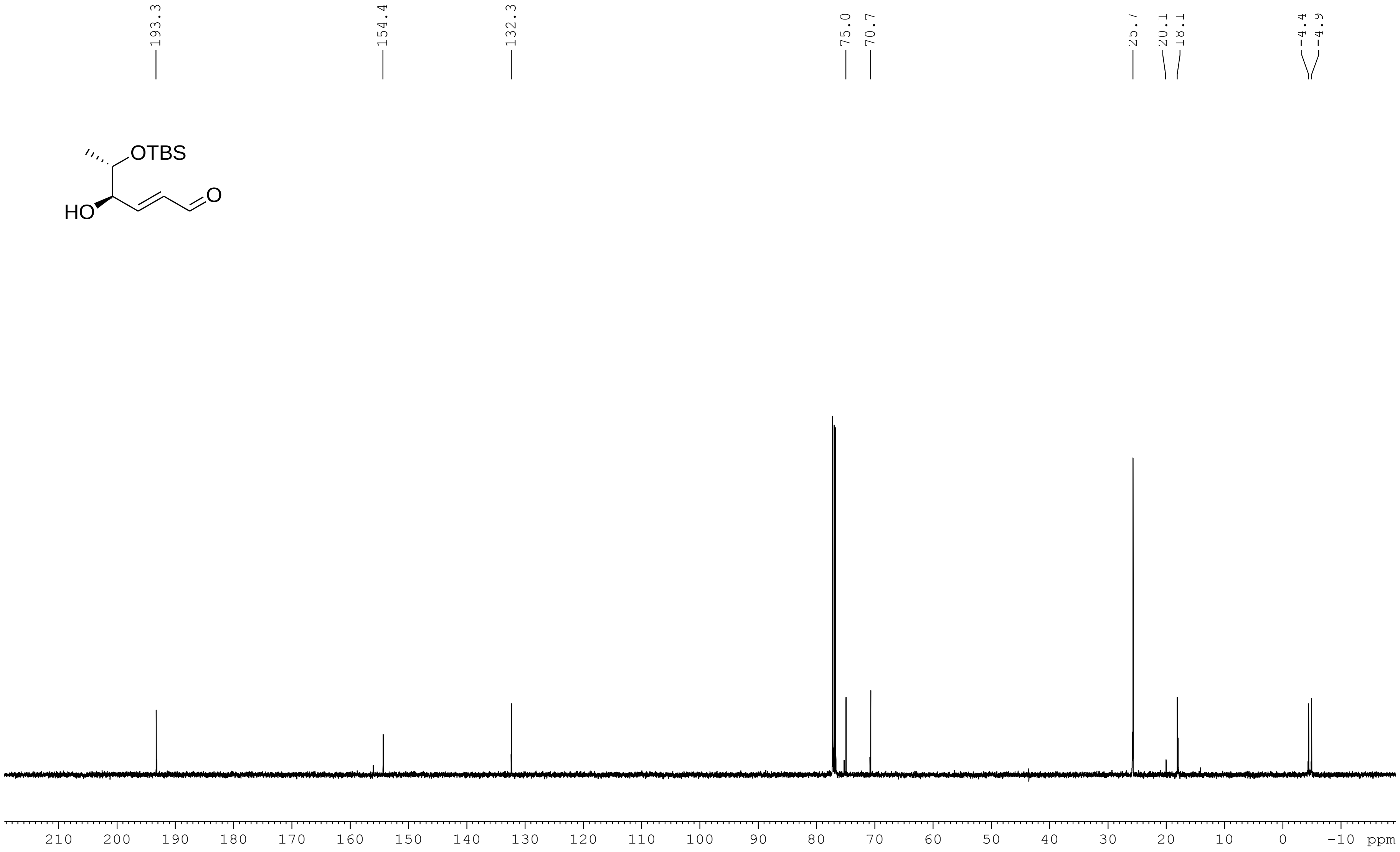

^{1}H NMR ($CDCl_3$, 300 MHz) of **9**

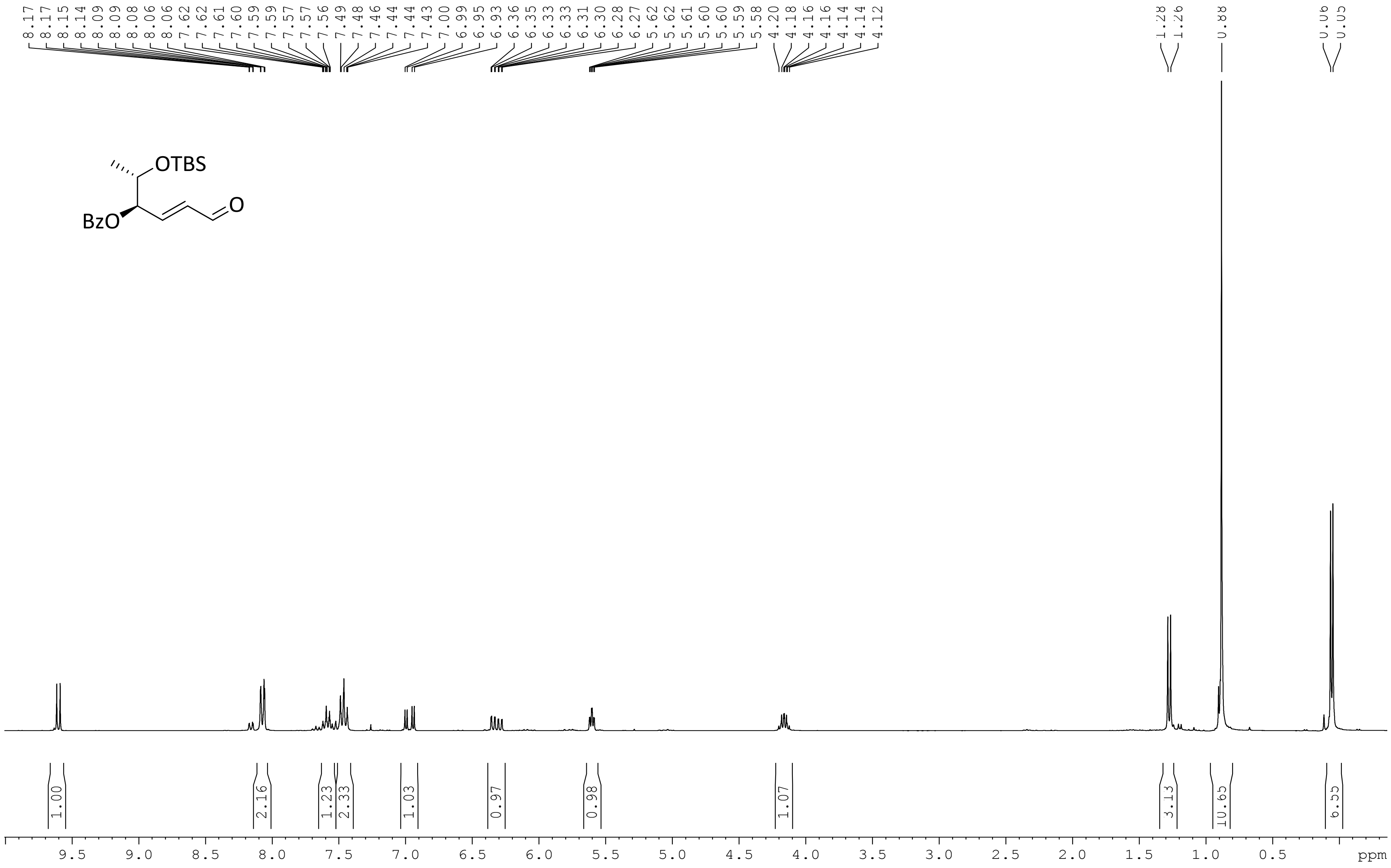

^{13}C NMR ($CDCl_3$, 75 MHz) of **9**

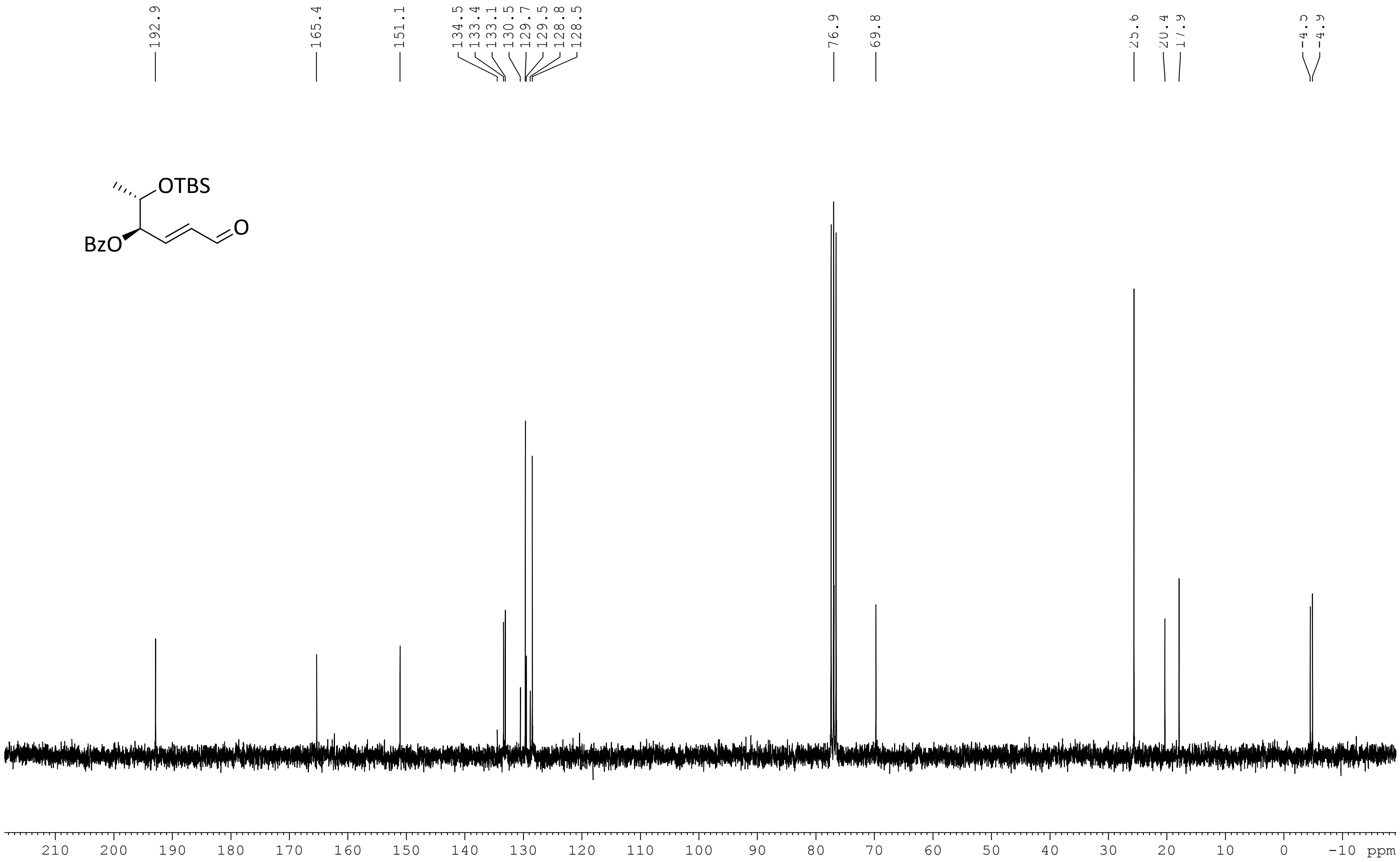

^{1}H NMR ($CDCl_3$, 300 MHz) of **10**

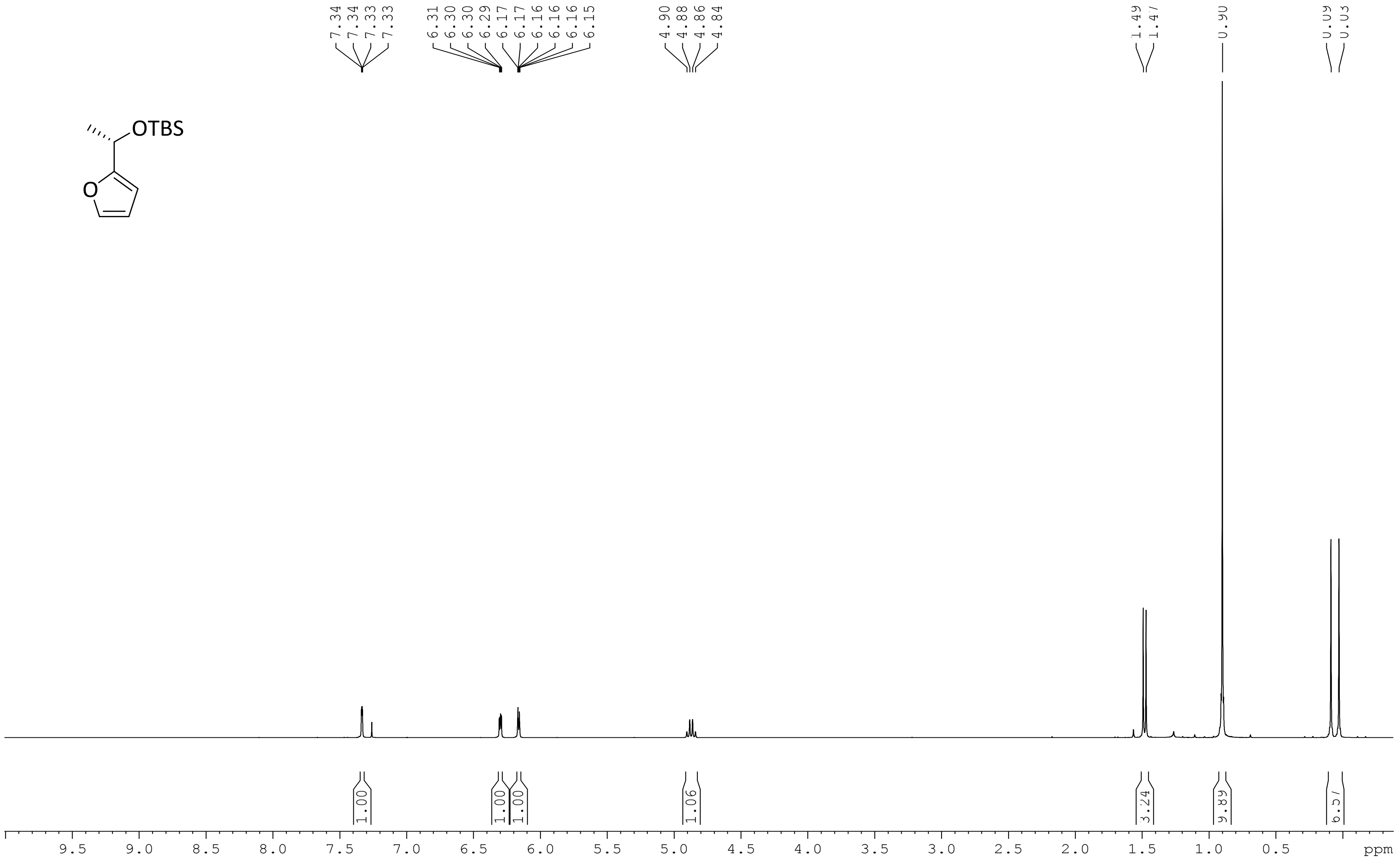

^{13}C NMR ($CDCl_3$, 75 MHz) of **10**

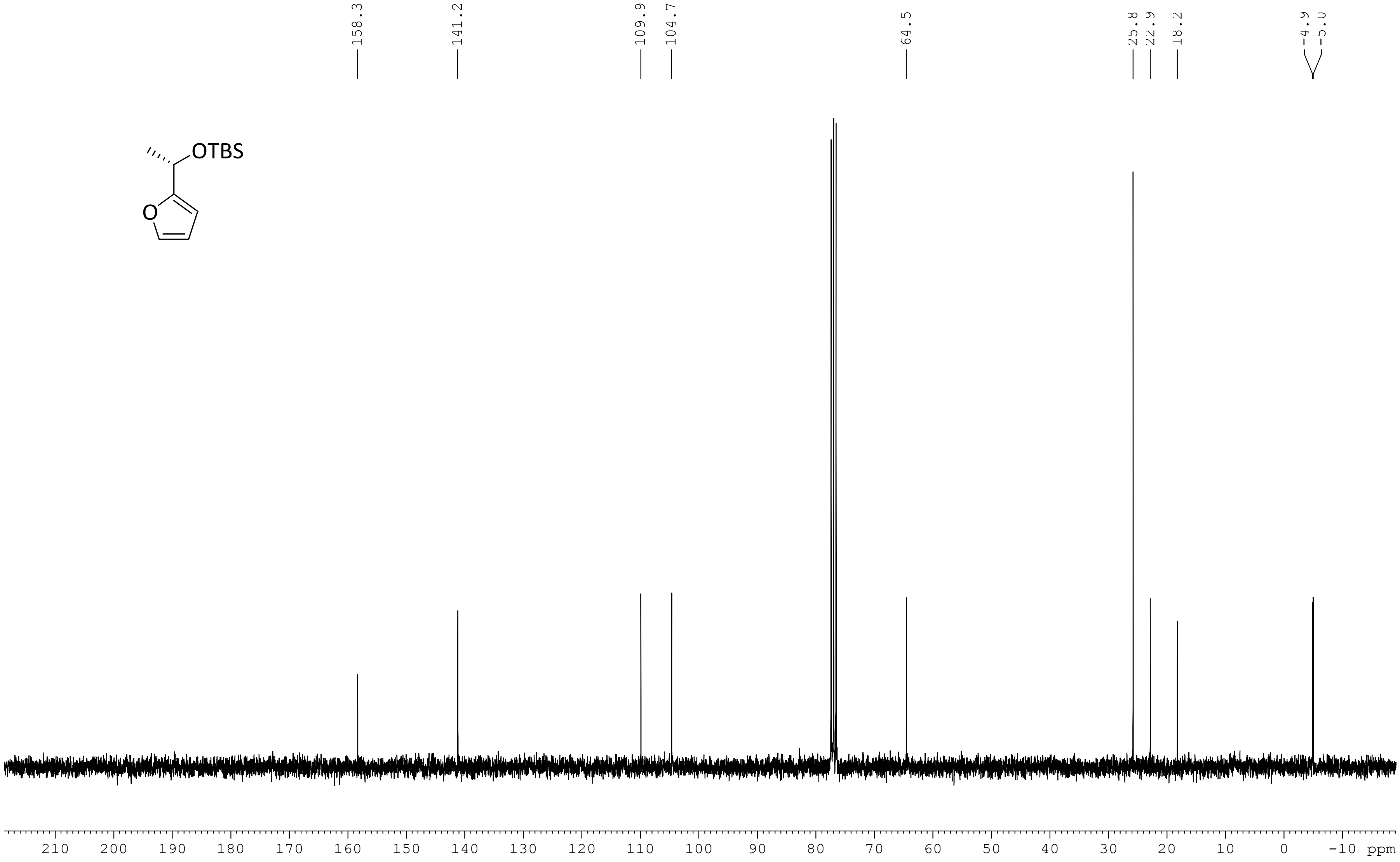

^{1}H NMR ($CDCl_3$, 300 MHz) of **11**

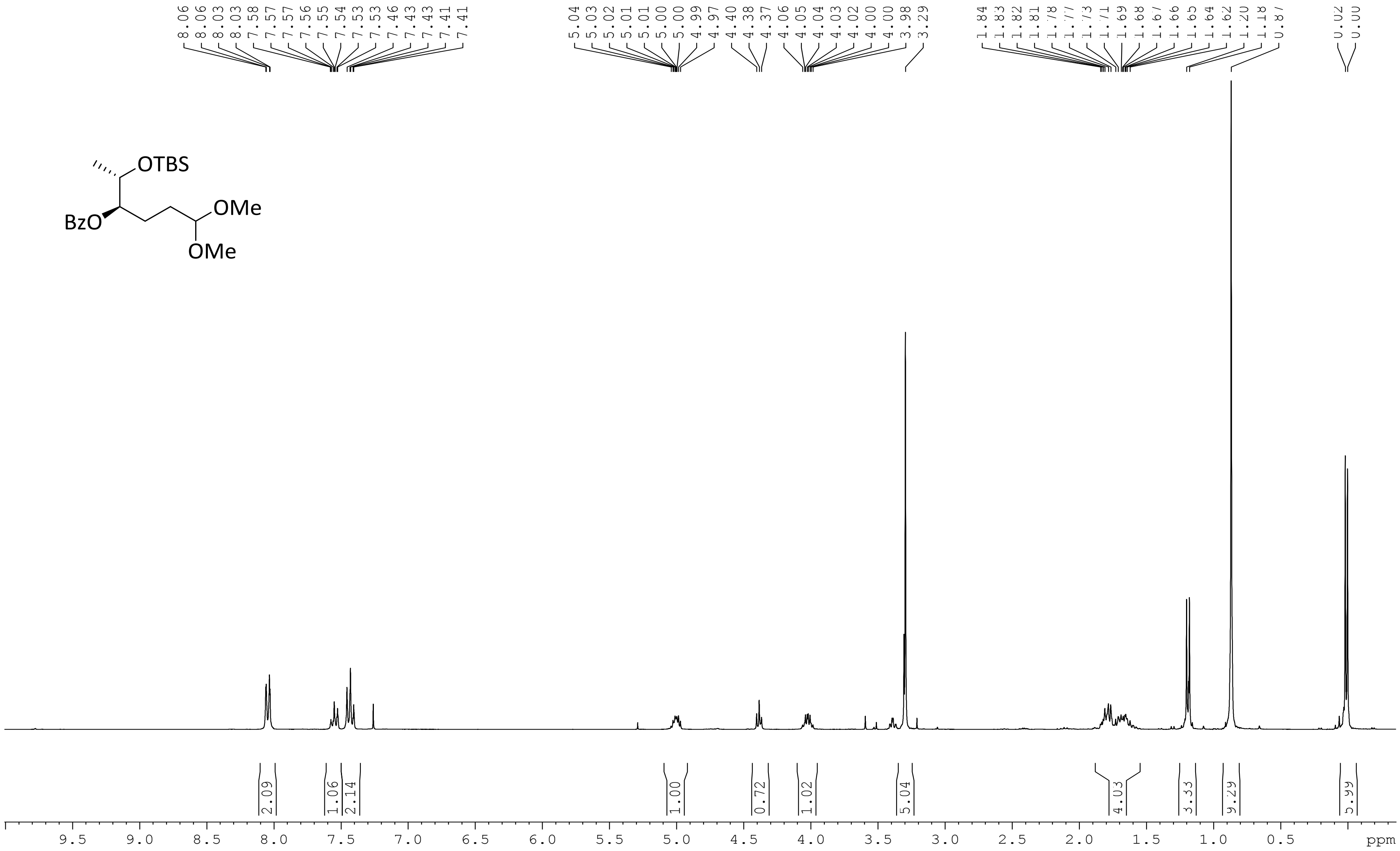

^{13}C NMR ($CDCl_3$, 75 MHz) of **11**

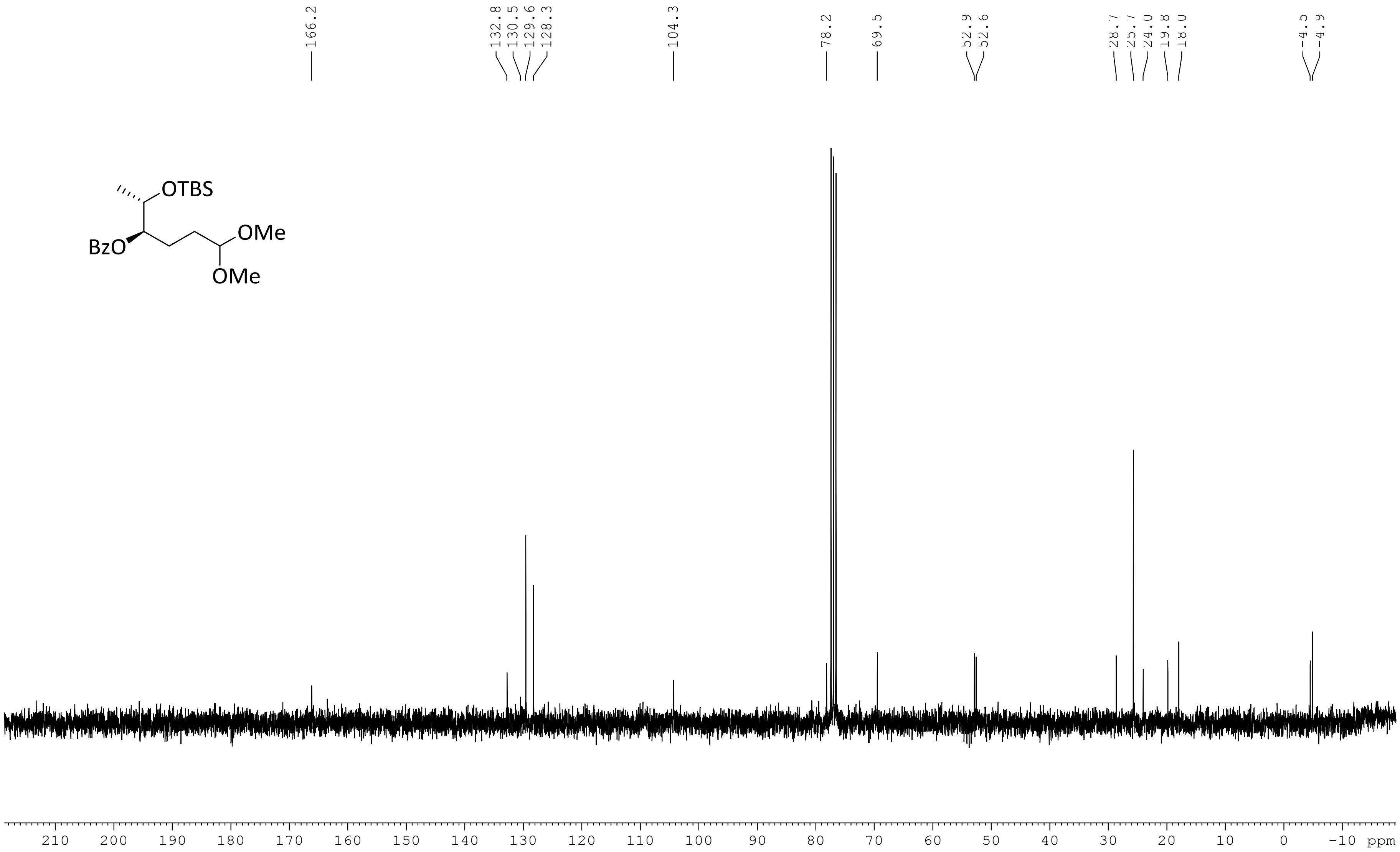

^{1}H NMR ($CDCl_3$, 300 MHz) for hydrogenation of enal **9** (inseparable mixture of **11** and **12**, see table 3, entry 3)

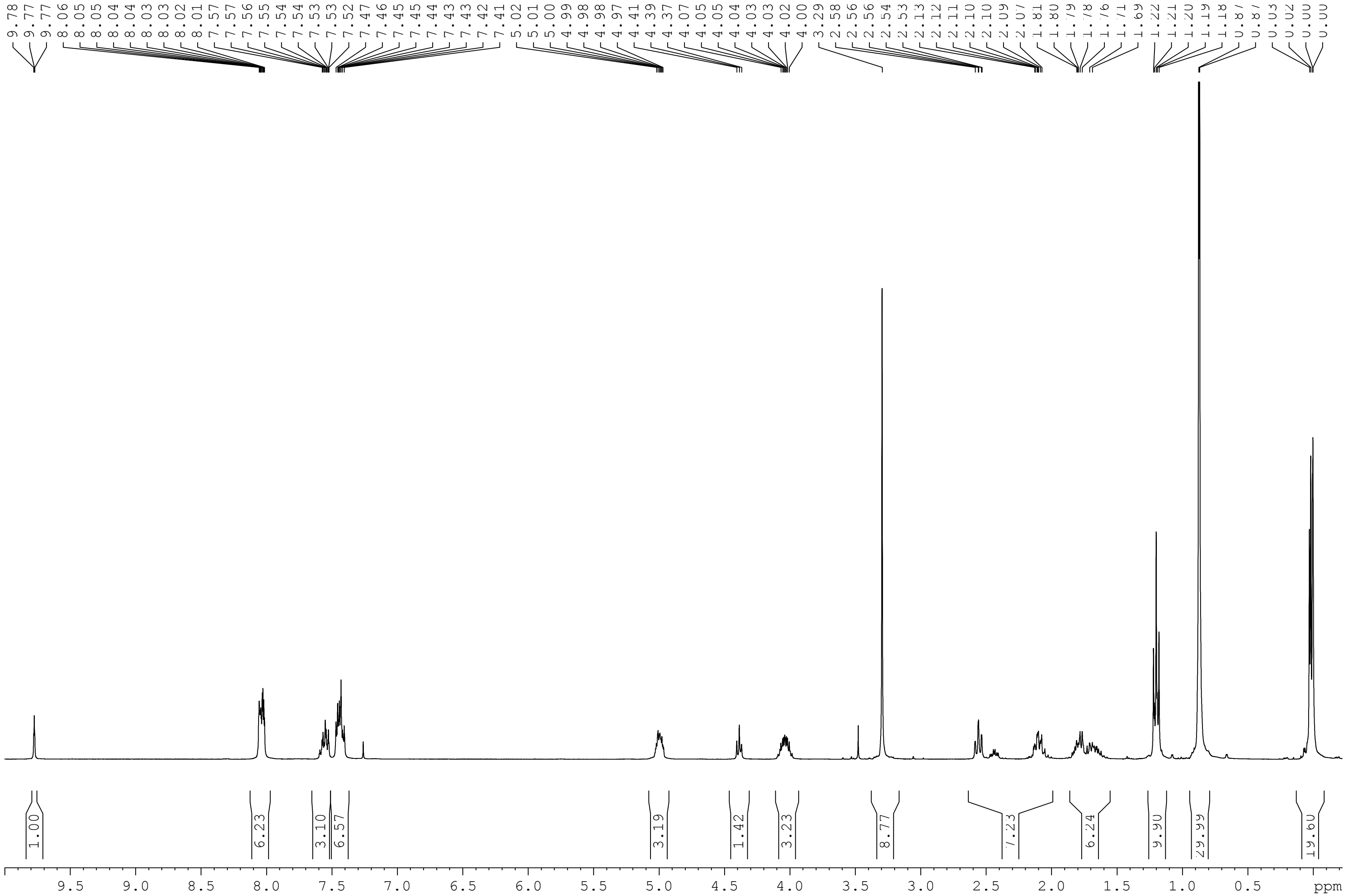

^{1}H NMR ($CDCl_3$, 300 MHz) of **13**

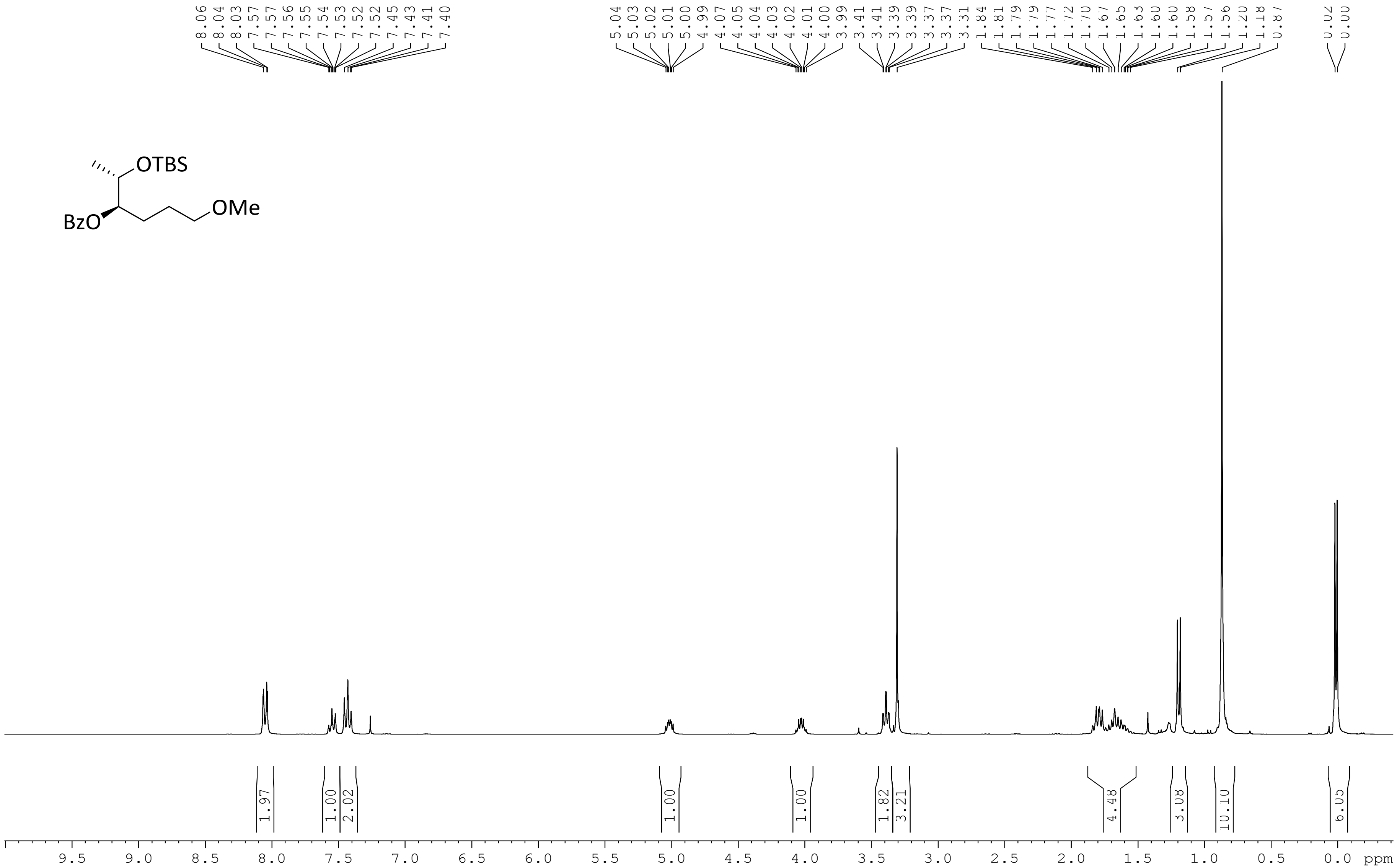

^{13}C NMR-APT ($CDCl_3$, 75 MHz) of **13**

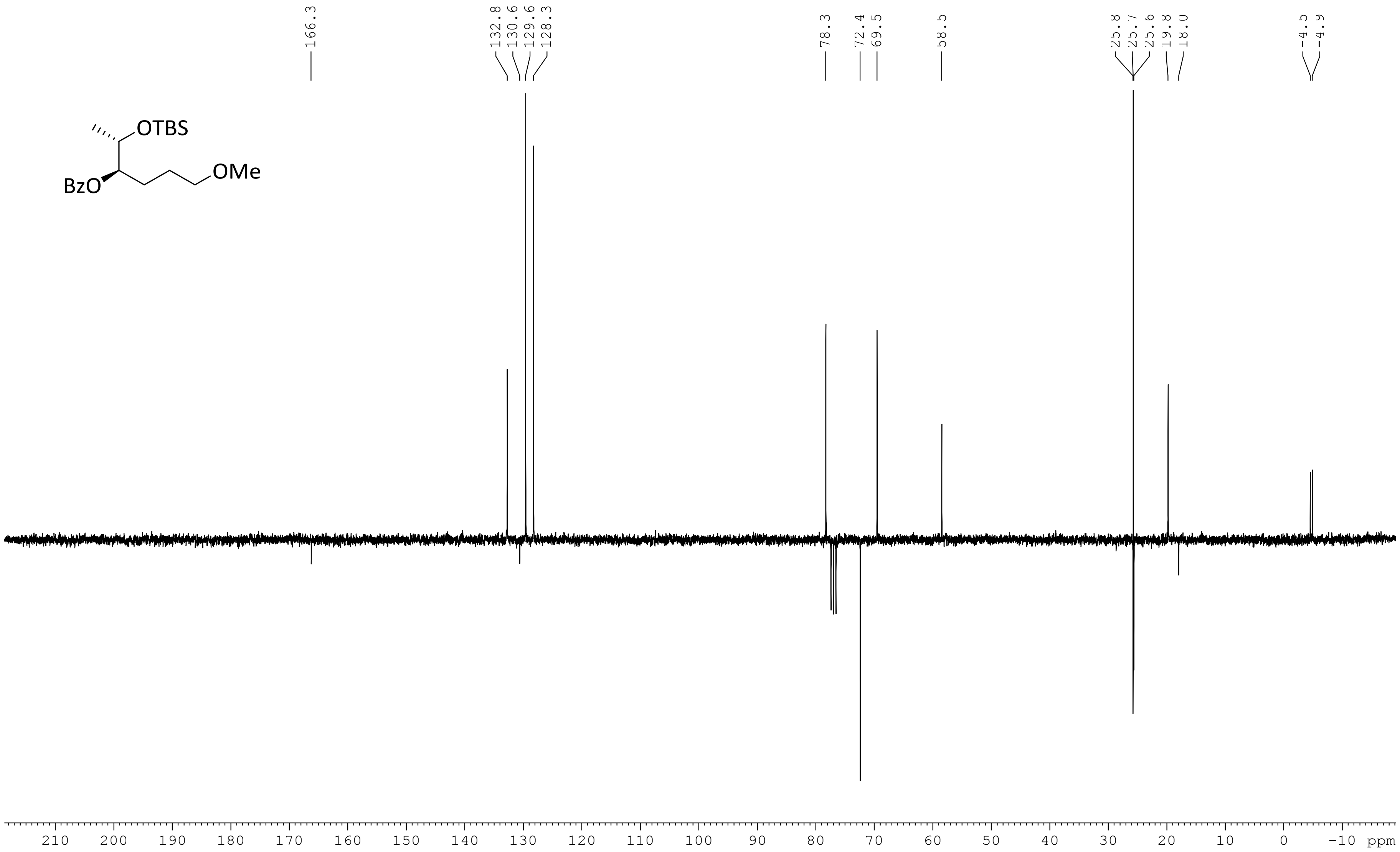

^{1}H NMR ($CDCl_3$, 300 MHz) of **16**

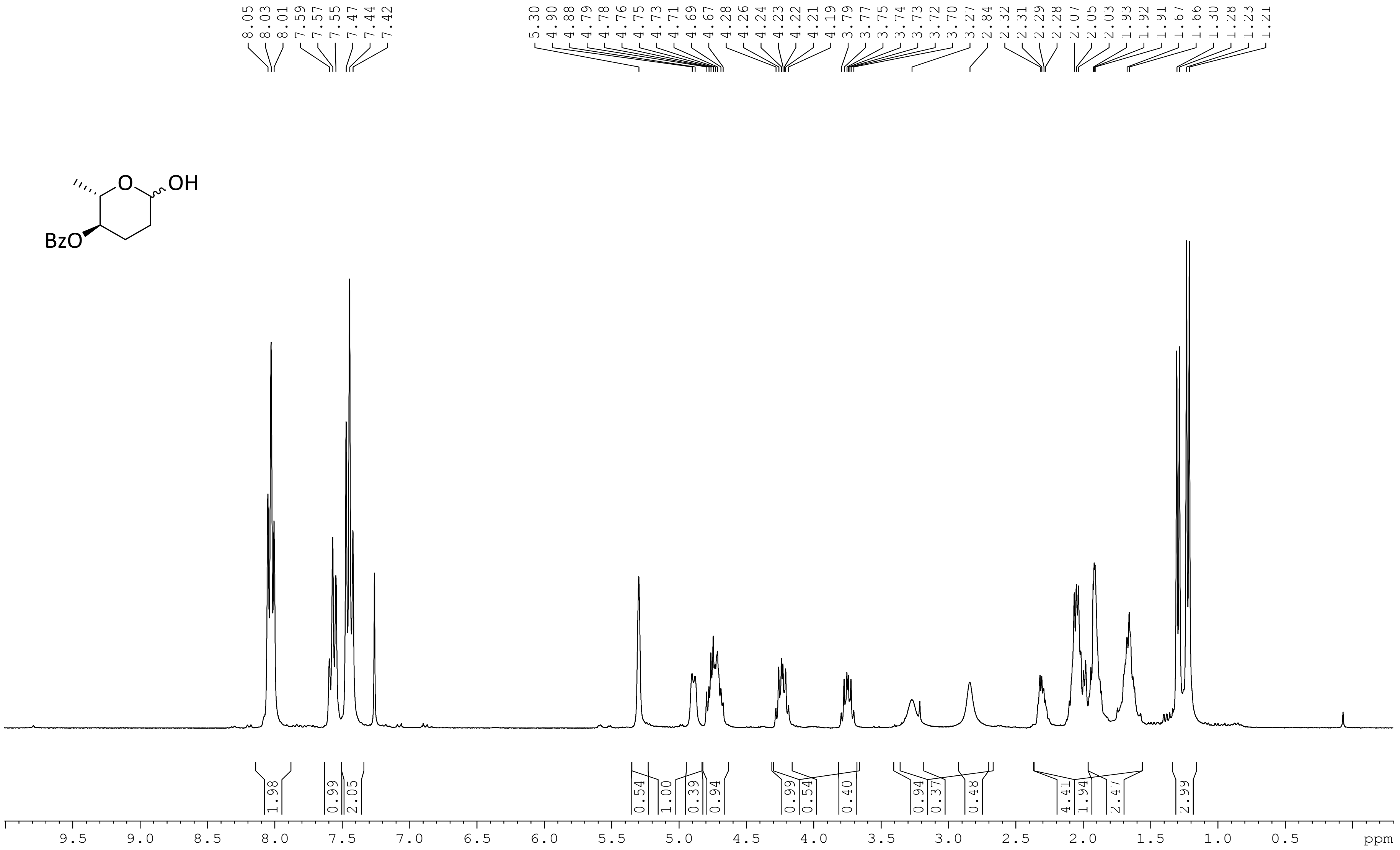

^{13}C NMR (CDCl$_3$, 75 MHz) of **16**

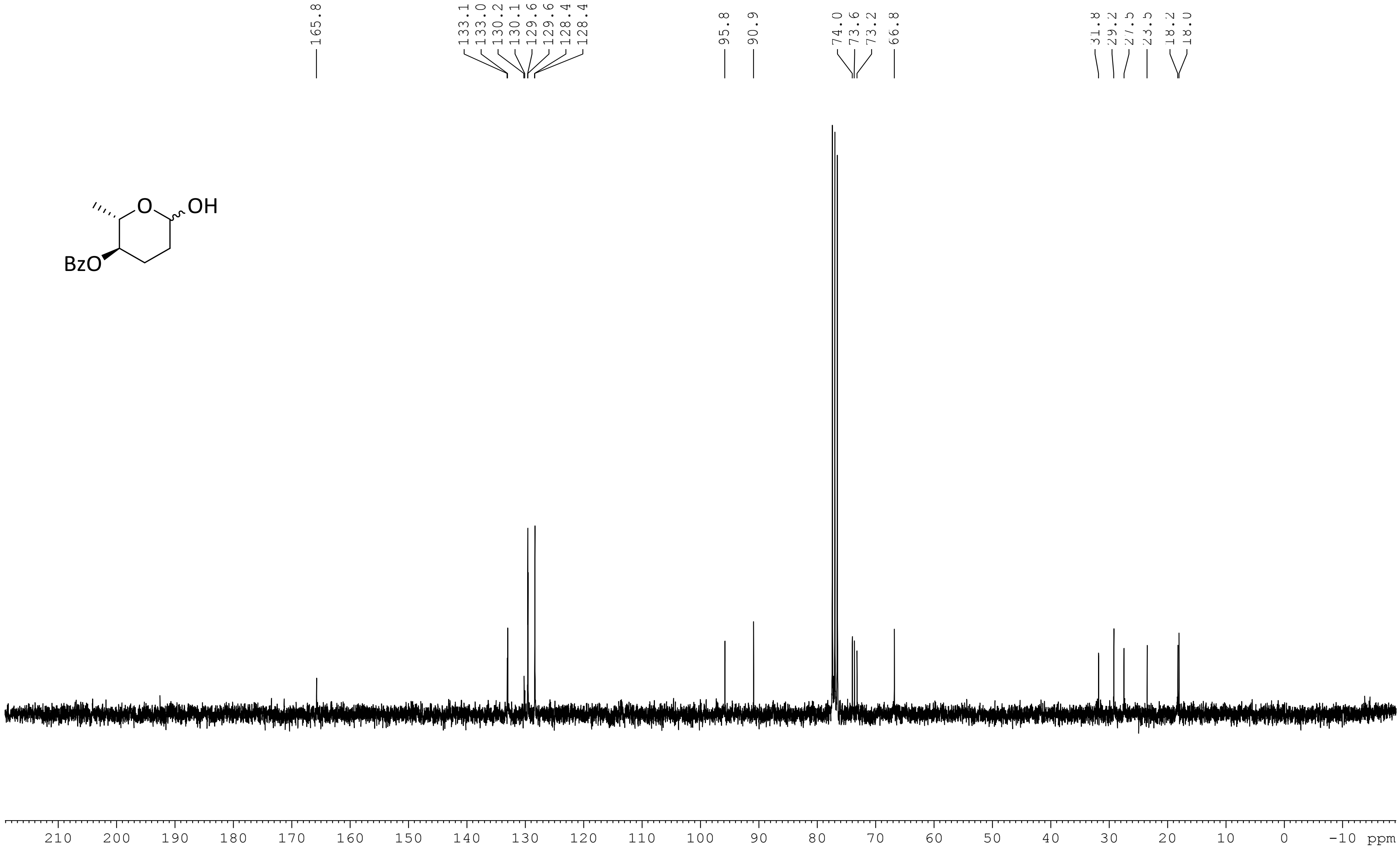

^{1}H NMR ($CDCl_3$, 300 MHz) of **17**

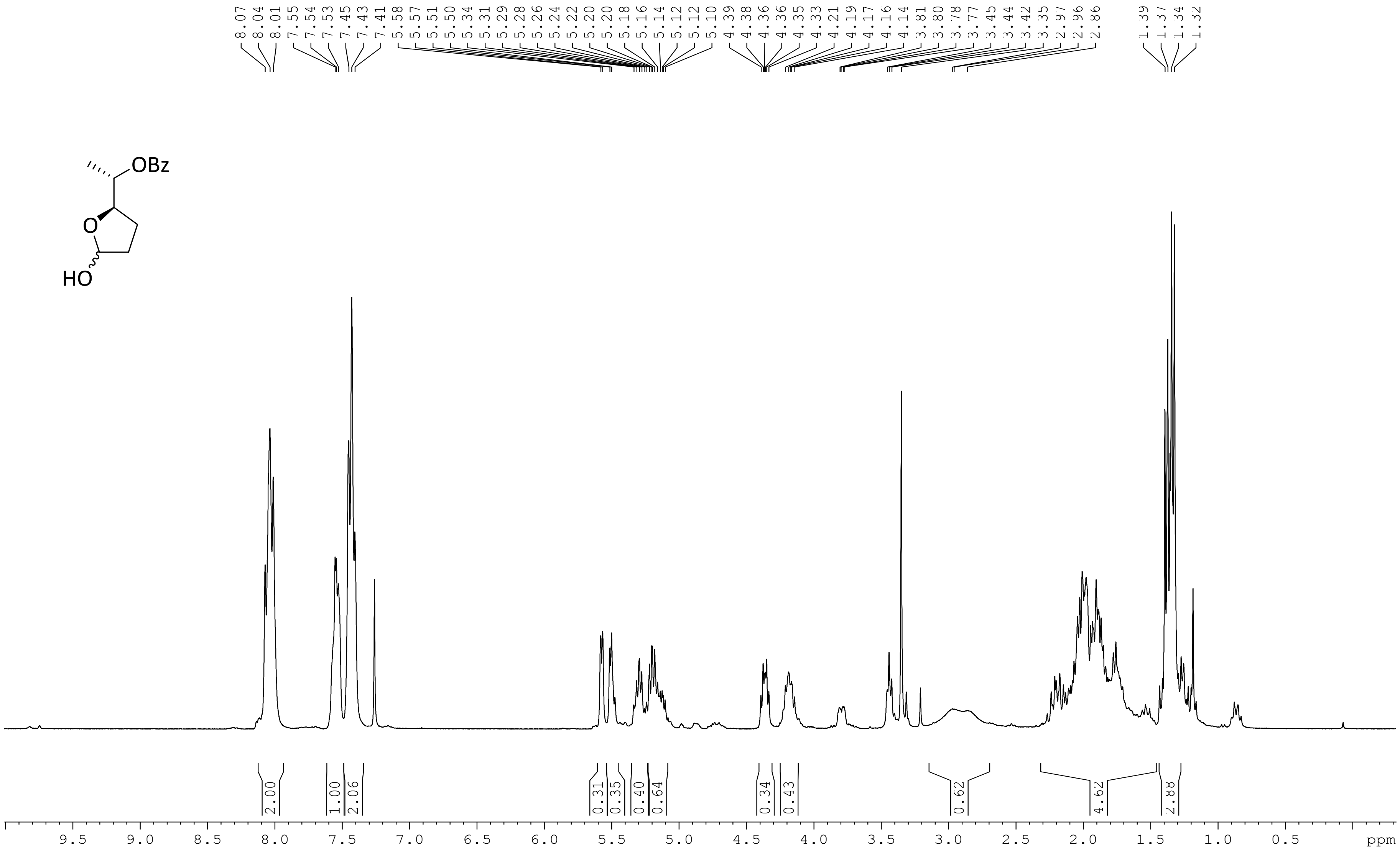

^{13}C NMR-APT ($CDCl_3$, 75 MHz) of **17**

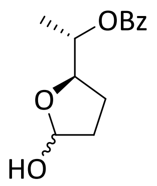

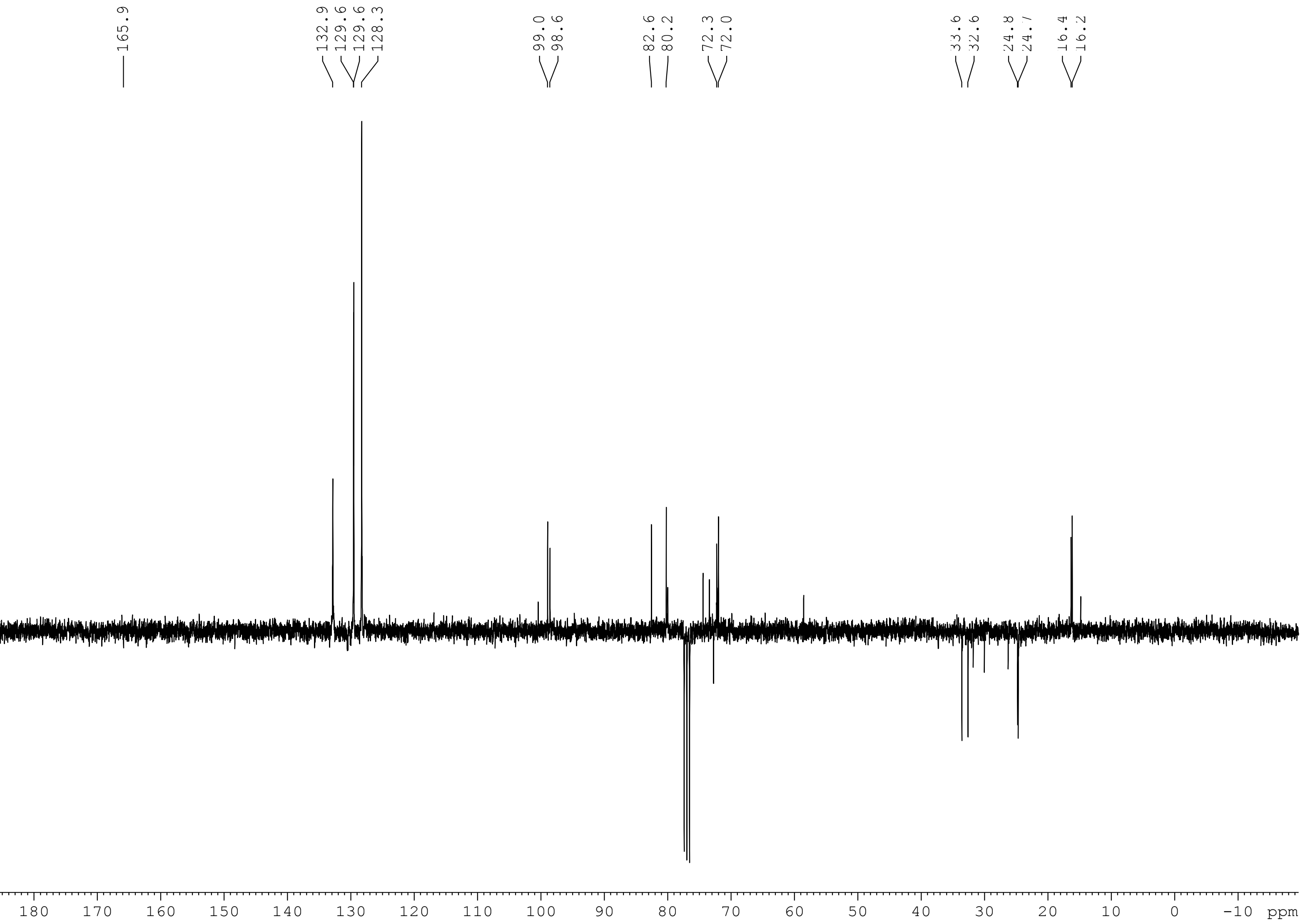

^{1}H NMR ($CDCl_3$, 300 MHz) of **21**

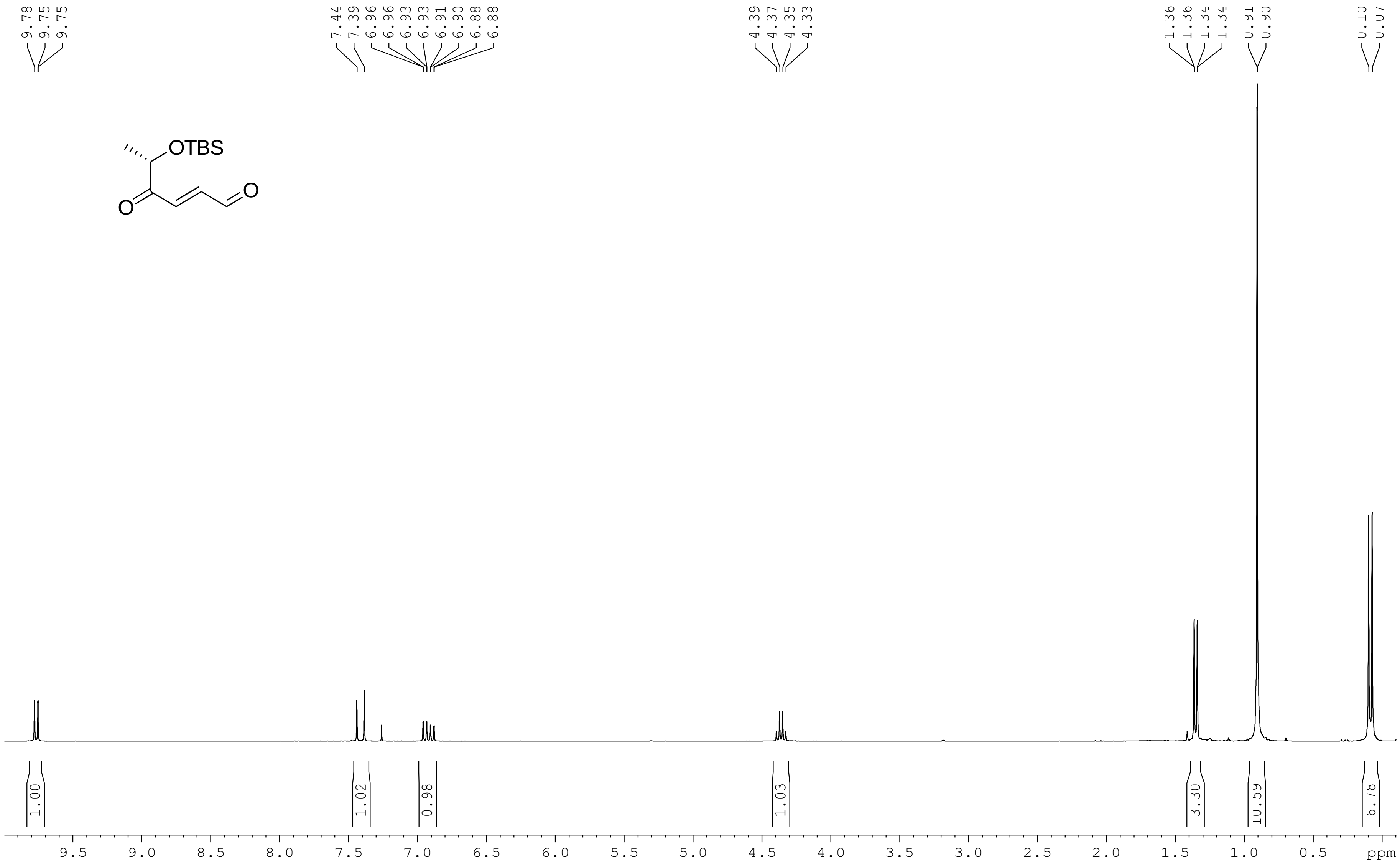

^{13}C NMR-APT ($CDCl_3$, 75 MHz) of **21**

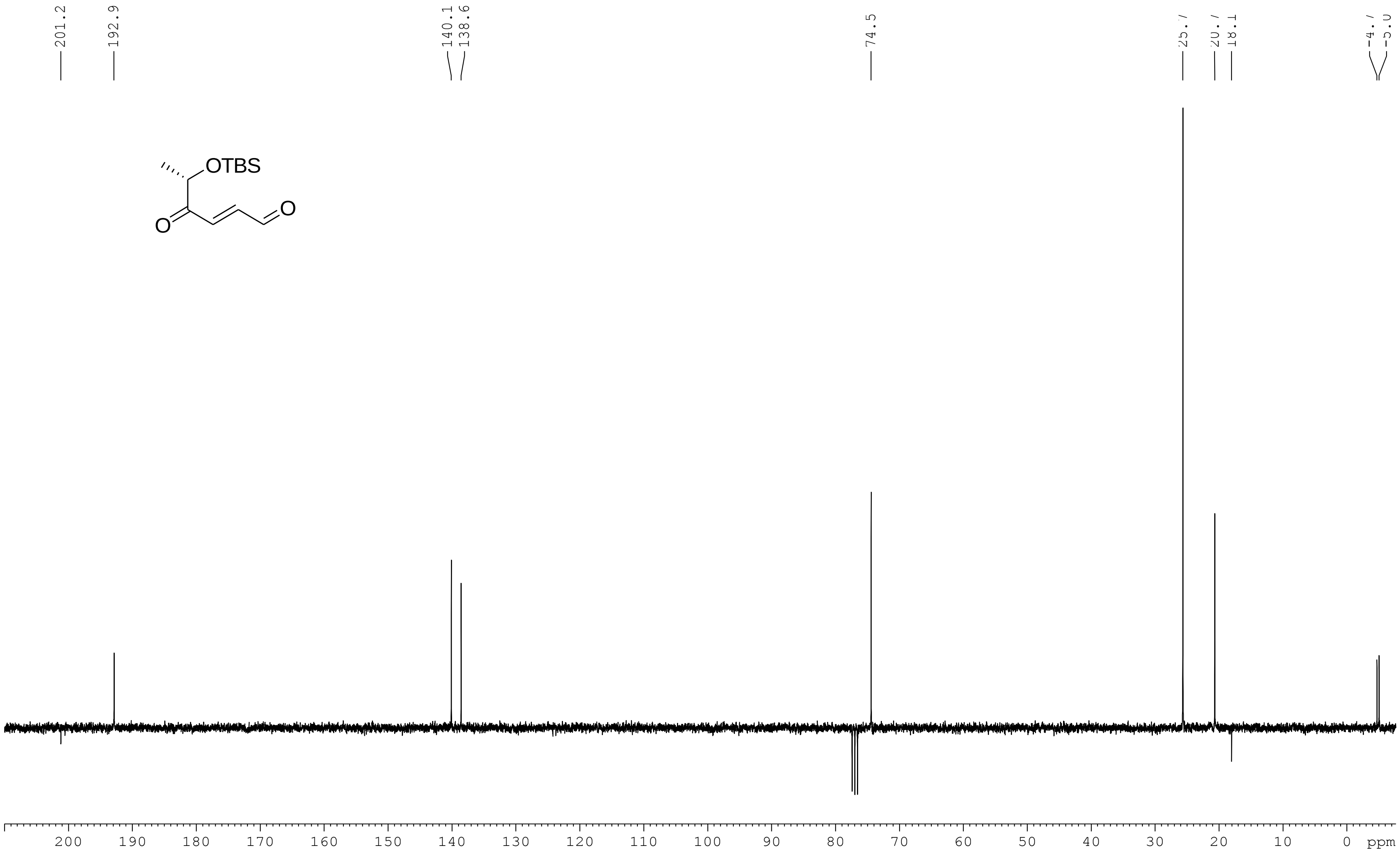

^1H NMR ($CDCl_3$, 300 MHz) of **22**

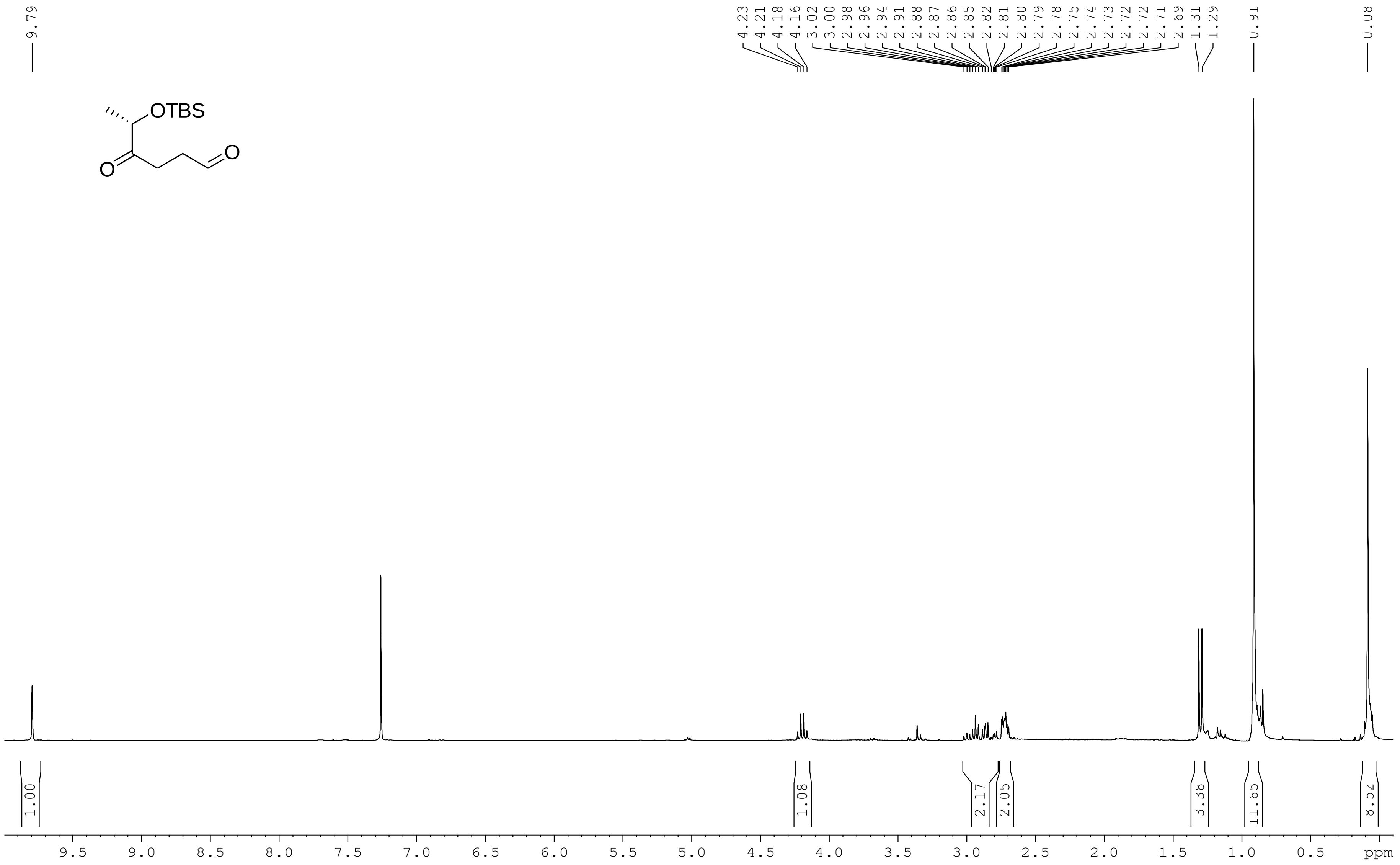

^{13}C NMR-APT ($CDCl_3$, 75 MHz) of **22**

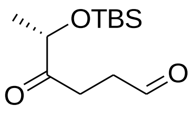

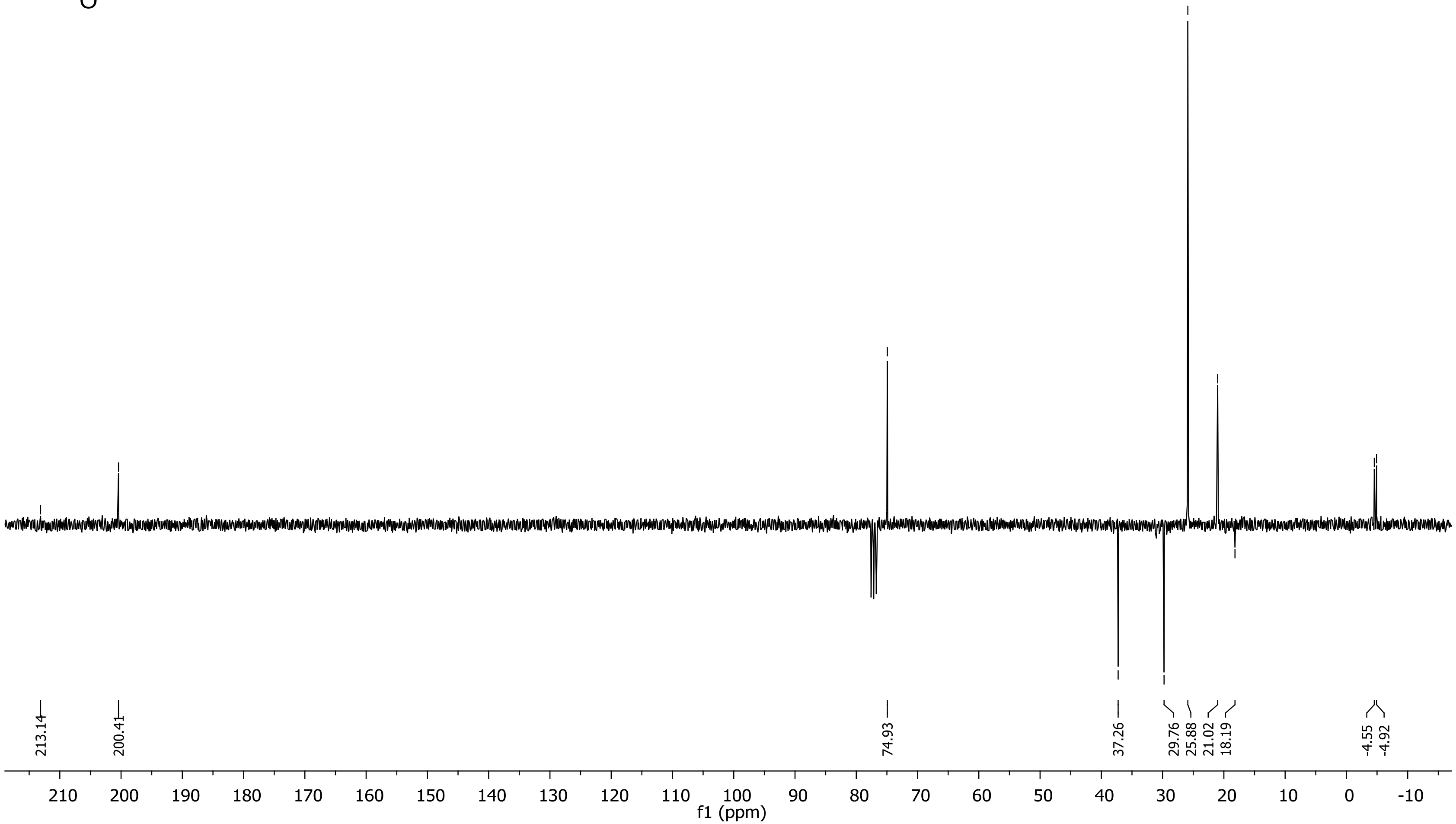

^{1}H NMR ($CDCl_3$, 300 MHz) of **23**

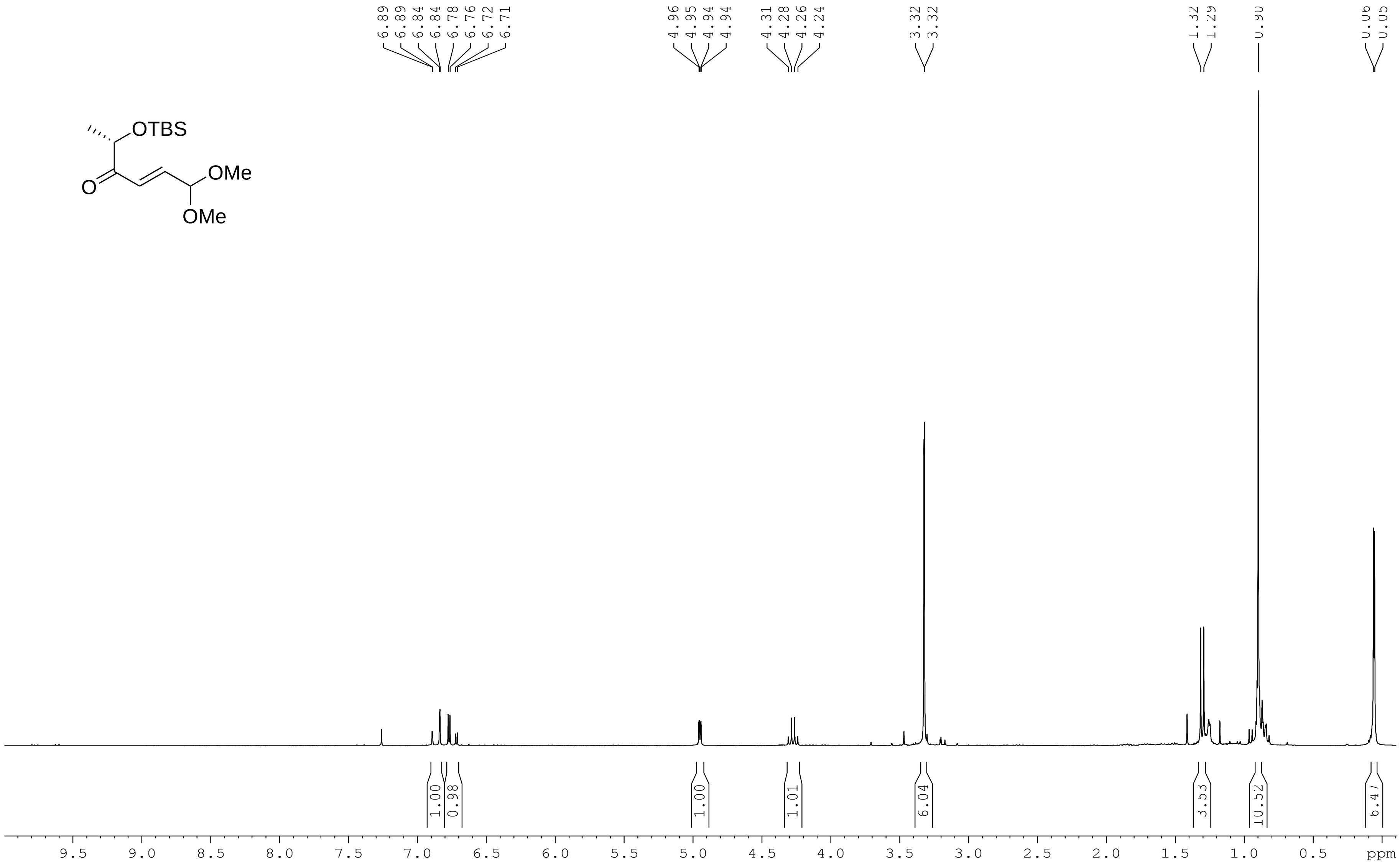

^{13}C NMR-APT ($CDCl_3$, 75 MHz) of **23**

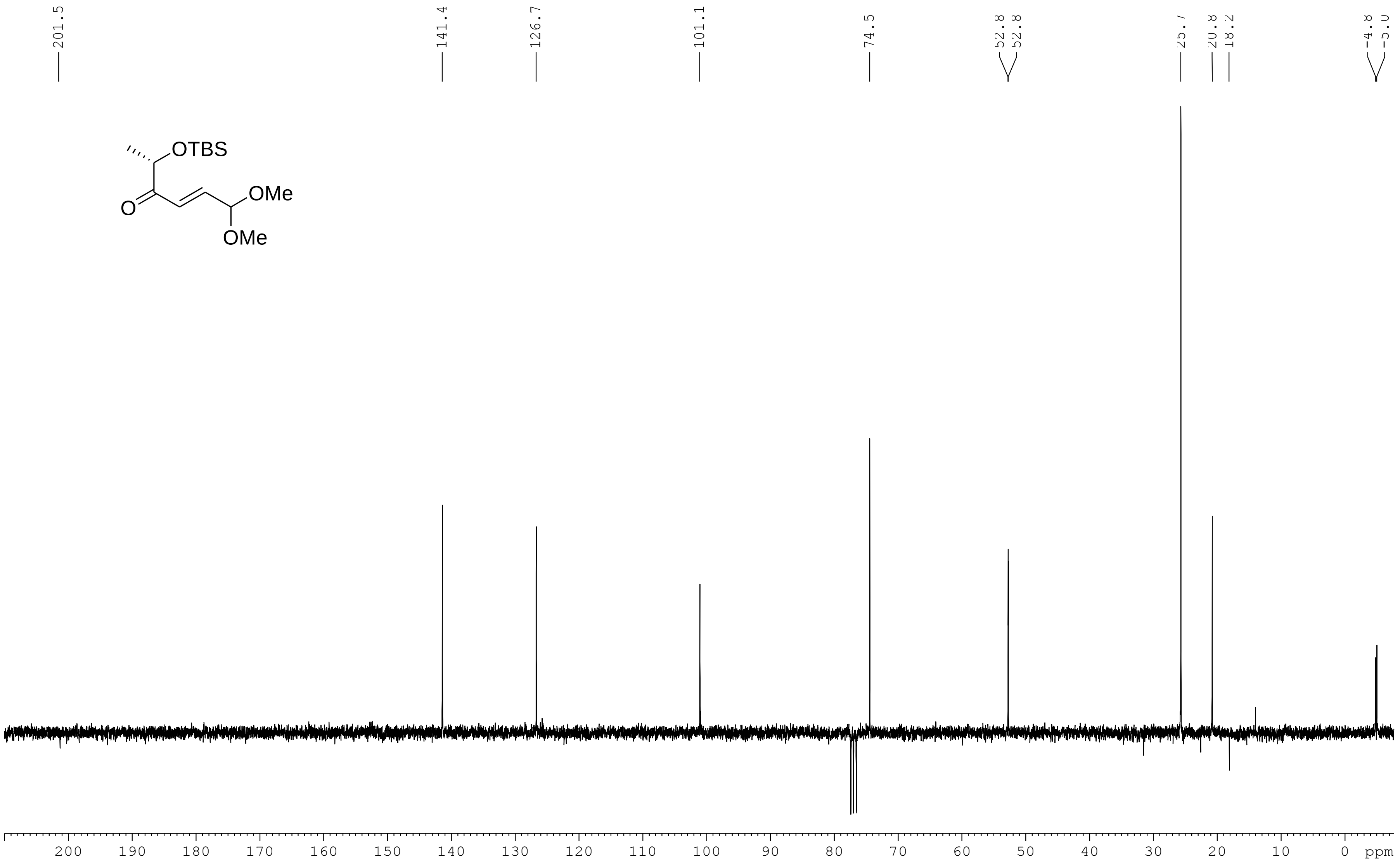

^{1}H NMR ($CDCl_3$, 300 MHz) of **24**

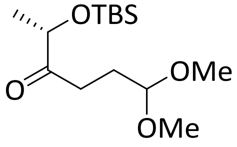

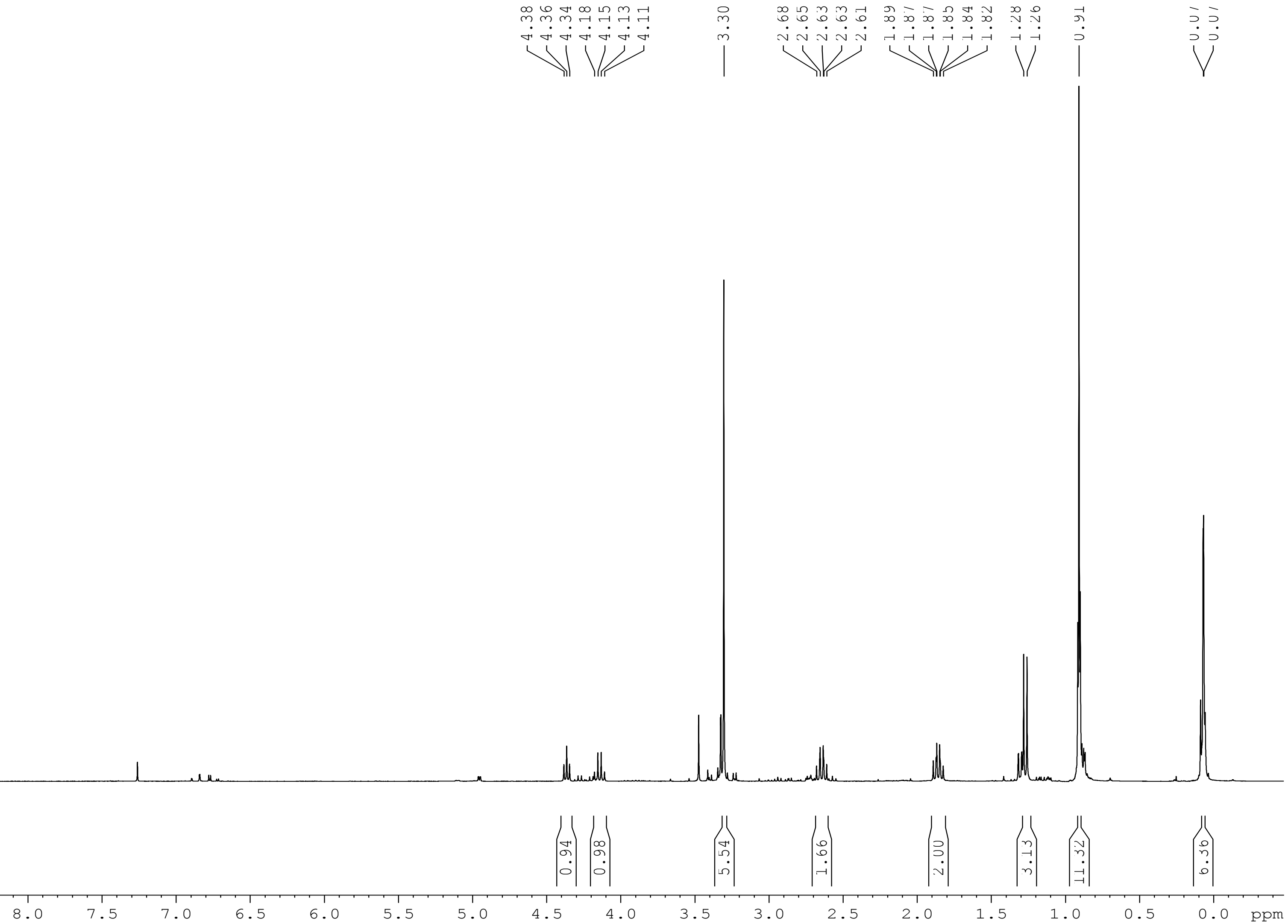

^{13}C NMR ($CDCl_3$, 75 MHz) of **24**

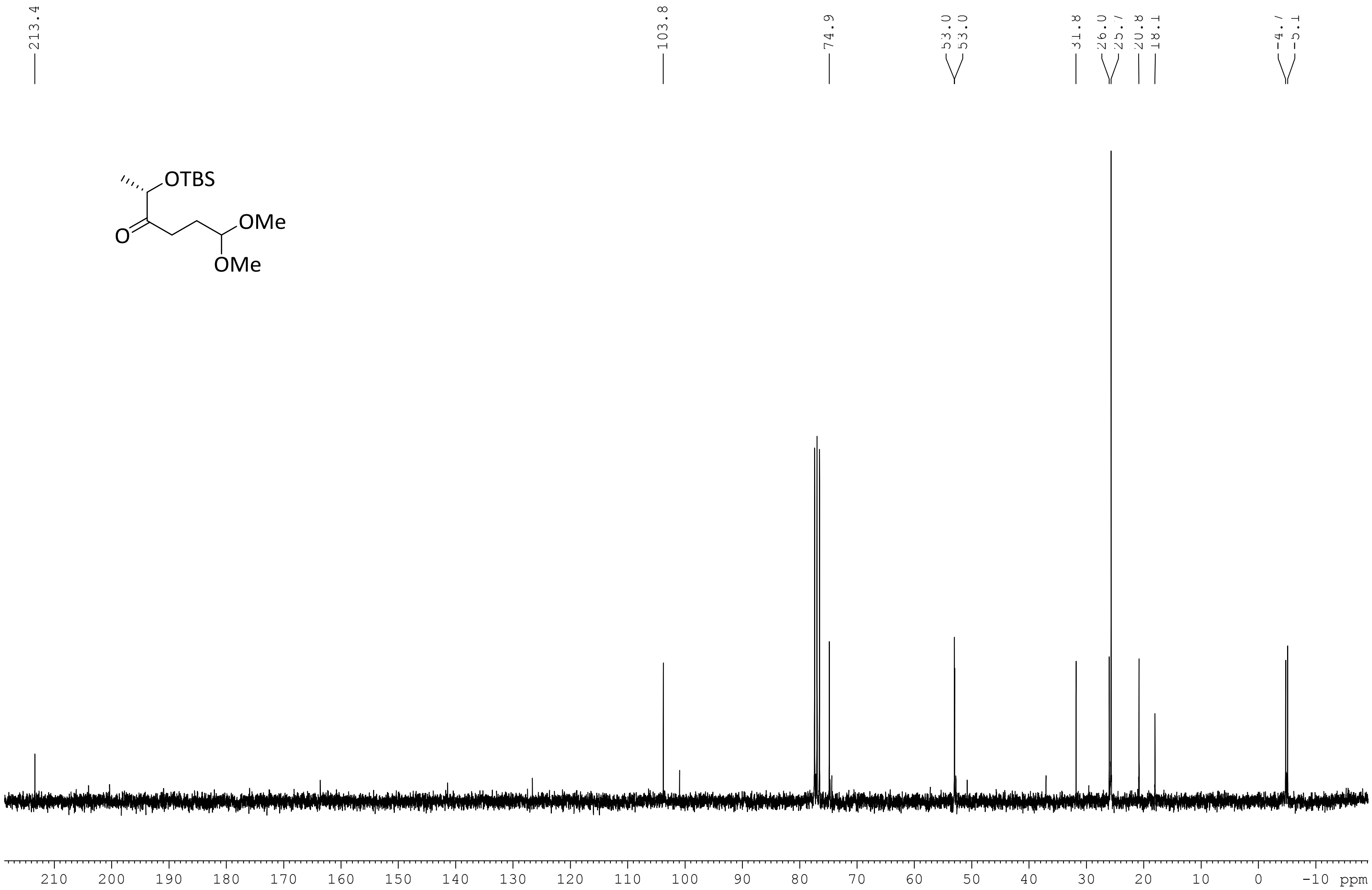

^{1}H NMR ($CDCl_3$, 300 MHz) for the mixture obtained from deprotection of **24**

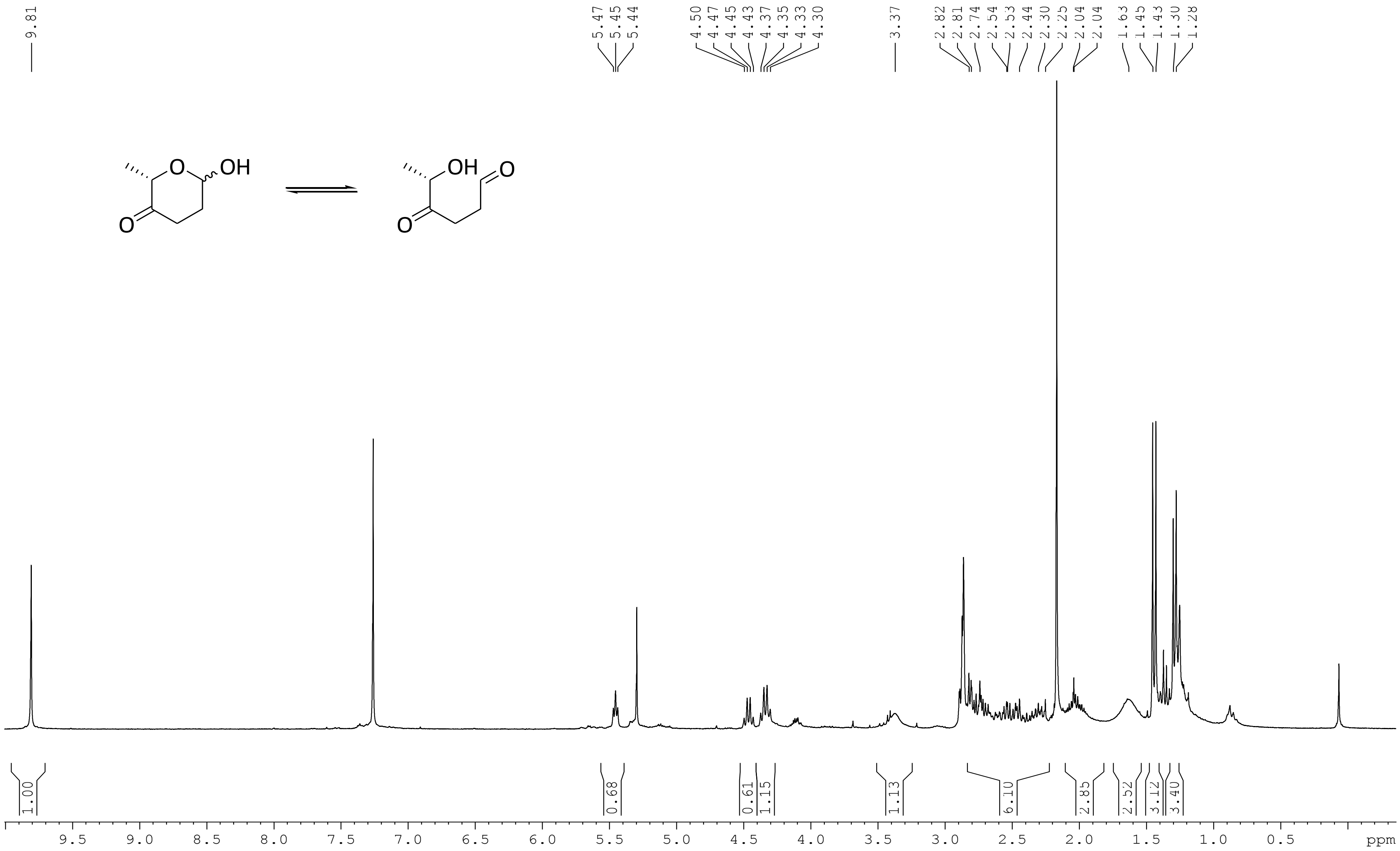

Supplement: File 2 — Copies of 1H and 13C NMR spectra. [file Beilstein_J_Org_Chem-10-1023-s002.pdf]
